# Methionine Mistranslation Bypasses the Restraint of the Genetic Code to Generate Mutant Proteins with Distinct Activities

**DOI:** 10.1371/journal.pgen.1005745

**Published:** 2015-12-28

**Authors:** Xiaoyun Wang, Tao Pan

**Affiliations:** Department of Biochemistry and Molecular Biology, University of Chicago, Chicago, Illinois, United States of America; University of Illinois, UNITED STATES

## Abstract

Although mistranslation is commonly believed to be deleterious, recent evidence indicates that mistranslation can be actively regulated and be beneficial in stress response. Methionine mistranslation in mammalian cells is regulated by reactive oxygen species where cells deliberately alter the proteome through incorporating Met at non-Met positions to enhance oxidative stress response. However, it was not known whether specific, mistranslated mutant proteins have distinct activities from the wild-type protein whose sequence is restrained by the genetic code. Here, we show that Met mistranslation with and without Ca^2+^ overload generates specific mutant Ca^2+^/calmodulin-dependent protein kinase II (CaMKII) proteins substituting non-Met with Met at multiple locations. Compared to the genetically encoded wild-type CaMKII, specific mutant CaMKIIs can have distinct activation profiles, intracellular localization and enhanced phenotypes. Our results demonstrate that Met-mistranslation, or “Met-scan” can indeed generate mutant proteins in cells that expand the activity profile of the wild-type protein, and provide a molecular mechanism for the role of regulated mistranslation.

## Author Summary

Methionine-mistranslation is a recently discovered phenomenon where mammalian cells deliberately mischarge non-Met-tRNAs with amino acid methionine in unstressed cells and in response to innate immune and chemically triggered oxidative stress. These mischarged tRNAs are used in translation to generate mutant proteins containing non-Met to Met substitutions. Accumulating evidence shows that cells employ regulated mistranslation to enhance response to oxidative and other stresses. However, it was unknown whether any specific mutant proteins generated in mistranslation truly have distinct activities as the wild-type protein. Here, we identify and characterize naturally occurring Met-mistranslated proteins in human cells and show that specific Met-mistranslated proteins can have very distinct properties compared to the wild-type protein *in vitro* and *in vivo*. For the Ca^2+^/calmodulin-dependent protein kinase II (CaMKII), Met-mistranslated CaMKII show significant differences in catalytic activities, subcellular localization and elevated caspase-3 activity from the wild-type CaMKII. Our results provide a molecular mechanism on how mistranslation is used as an adaptive mechanism to stress, and establish Met-mistranslation as a nature’s way to overcome the restraint on the protein sequence by the genetic code.

## Introduction

Although it was commonly believed that translational fidelity is maintained at high levels in cells at all times, accumulating evidence indicates that mistranslation is also regulated in cells, and mistranslation may broaden proteomic and phenotypic diversity to help cells respond to stress [1,2,3,4,5]. Regulated mistranslation through tRNA misacylation in mammalian cells is among the best studied case so far in advancing our understanding on how mistranslation may also be beneficial. Met-mistranslation is derived from misacylating non-methionyl-tRNAs with the amino acid methionine. In mammals, Met misacylation is regulated by reactive oxygen species (ROS), can vary over a wide range [1], and is controlled through ERK phosphorylation of two Ser residues in the methionyl-tRNA synthetase (MetRS, [6]). Constitutive expression of the inaccurate (Ser-to-Asp) MetRS mutant in cells results in enhanced response to oxidative stress; conversely, constitutive expression of the accurate (Ser-to-Ala) MetRS mutant in cells results in decreased stress response [6]. Numerous non-Met to Met-substituted mutant proteins have been identified by mass spectrometry [1,6,7] or through specifically designed fluorescent protein [6], indicating that Met misacylated tRNAs are directly used in translation. Presumably, the cellular phenotype conferred by Met-mistranslation is exerted through specific mutant proteins. However, it was not known whether any of the mutant proteins generated in Met-mistranslation has distinct activities compared to the wild-type protein.

Ca^2+^/calmodulin-dependent protein kinase II (CaMKII) is a multifunctional protein necessary for cellular Ca^2+^ homeostasis [8]. The CaMKII holoenzyme is assembled from 12 subunits, each subunit contains three key domains: the catalytic domain which performs the kinase function, the regulatory domain which controls enzyme activation and autoinhibition, and the association domain which directs its multimeric assembly [8]. CaMKII can be activated by calcium/calmodulin (Ca^2+^/CaM) to regulate signaling cascades in various cellular processes [8], and excessive activation of CaMKII causes apoptosis in a caspase-3-dependent pathway [9]. CaMKII activity can also be alternatively regulated by ROS through oxidation of two genetically encoded Met residues, and thus CaMKII has been conceived as a key ROS sensor [9,10]. Misregulation of CaMKII activity is closely linked to ROS-dependent diseases in heart and brain and in diabetes [11,12,13]. For instance, it has been demonstrated that exogenous Ca^2+^ influx could trigger translocation or redistribution of CaMKII to different subcellular compartments where CaMKII implements its biological roles with target molecules [14,15,16,17].

**Fig 1:**
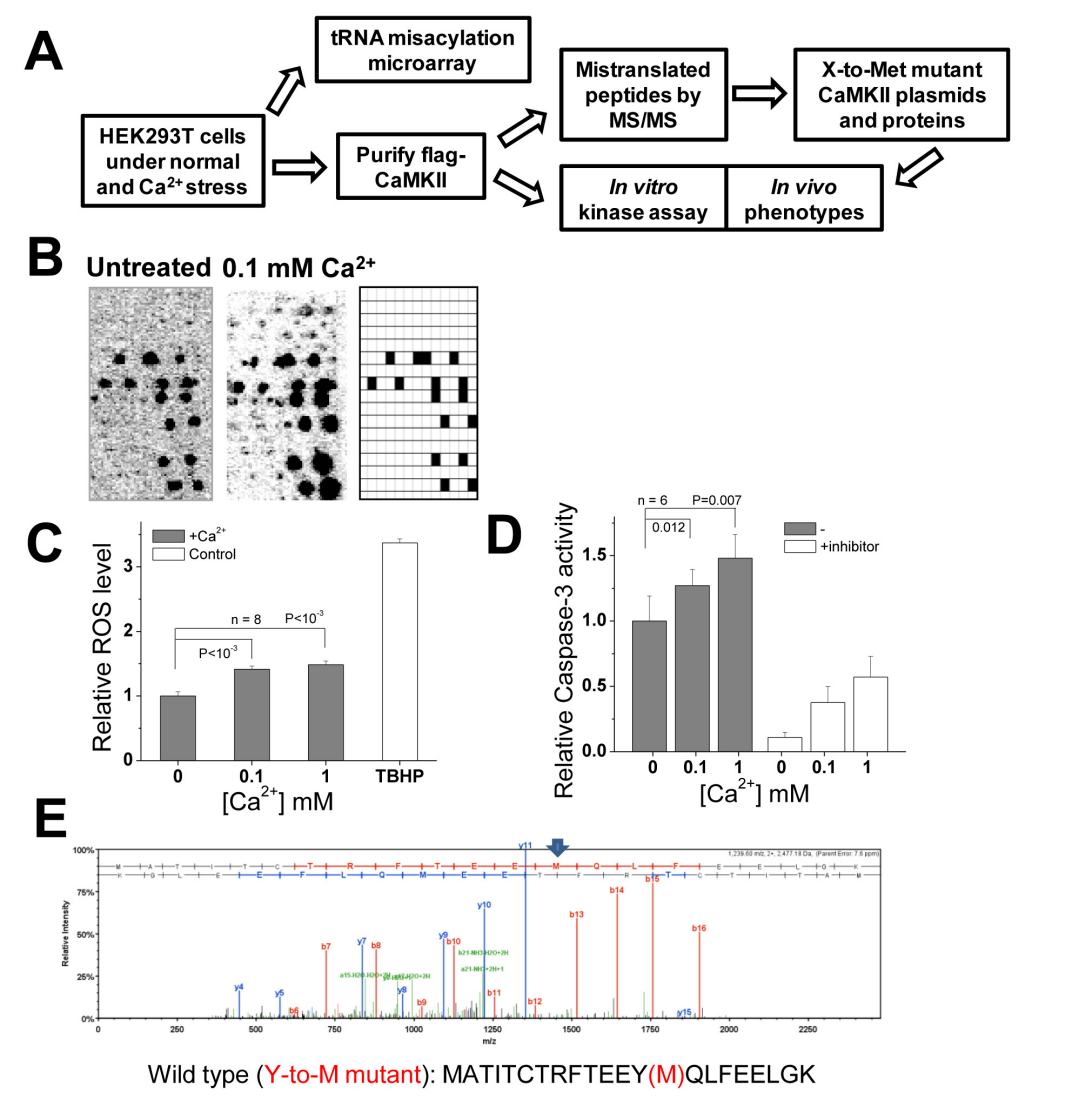
Ca^2+^ stress induces tRNA met-misacylation and identification of Met-mistranslated CaMKII proteins. (**A**) Flow diagram summarizing the experiments and goals of this work. (**B**) tRNA misacylation with methionine measured by tRNA microarrays in the absence or presence of Ca^2+^ stress. Like many chemical inducers demonstrated earlier [1], Ca^2+^ stress also increased tRNA misacylation. The array layout on the right shows the location of the Met-tRNA probes. (**C**) Cellular ROS measurement. HEK293T cells were suddenly exposed to Ca^2+^ at different concentrations (0, 0.1 or 1 mM) for 16 h, and cellular ROS level was measured. Tert-butyl hydrogen peroxide (TBHP) was used as positive control; n = 8 biological replicates. (**D**) Caspase-3 activity upon addition of 0.1 or 1 mM Ca^2+^. Z-DEVD-AMC was used as the substrate and Ac-DEVD-CHO was used as the inhibitor to confirm caspase-3 activity; n = 6 biological replicates. In panels (C) and (D), data are expressed as Mean ± S.D. of independent replicates, data between groups are compared by Student's t test and the P values are shown. (**E**) A Met-mistranslated CaMKII peptide identified by mass spectrometry. The Tyr-to-Met conversion is indicated by an arrow, and the peptide sequence is shown in the left side of the spectrum.

Here, we investigate whether Met-mistranslation indeed generates specific mutant proteins that have distinct activities as the genetically encoded, wild-type protein. Inspired by our findings that a sudden Ca^2+^ influx also triggers higher level of tRNA misacylation in a human cell line, we used CaMKII protein as a model system to address the question of distinctly mistranslated, mutant protein activities and to elucidate whether Ca^2+^ stress-mediated Met-mistranslation can have biological consequences on this important protein involved in Ca^2+^ signaling (Fig 1A). We identify specific Met-mistranslated proteins from HEK293T cells by mass spectrometry in the presence and absence of Ca^2+^ stress. Biochemical characterizations of the wild-type and mutant proteins derived from cellular Met-mistranslation show that specific mutant CaMKII proteins have significantly different kinase activation profiles and autophosphorylation levels. In addition, specific mutant CaMKII proteins show different levels in cellular compartments and corresponding differences in the caspase-3 activity triggered by the Ca^2+^ stress. Our results provide direct evidence that Met-mistranslation, or “Met-scan” of the wild-type, genetically encoded protein can indeed broaden the activity profile of cellular proteins.

## Results

### Ca^2+^ stress induces tRNA met-misacylation

We first investigated whether Ca^2+^ stress also induced tRNA misacylation using a previously established microarray method (Fig 1B) [1]. We found previously that tRNA misacylation had a low basal level in unstressed mammalian cells, and was further induced through innate immune activation by viruses, double-stranded RNA or polyliposaccharide, or by chemicals such as H_2_O_2_ or arsenite that trigger oxidative stress. Here, we found that higher level of tRNA misacylation was also induced by a sudden addition of Ca^2+^ directly to the growth medium after cells have been growing for 48 hr, even though the growth medium already contains substantial amount of Ca^2+^ (Fig 1B).

Ca^2+^ overload is known to trigger elevated ROS levels [18,19,20]. To uncover the reason for tRNA misacylation induced by sudden addition of Ca^2+^, we measured cellular ROS level upon treatment (Fig 1C). Indeed, sudden exposure of mammalian cells with 0.1 mM or 1 mM Ca^2+^ increased cellular ROS levels by 40%-50% (*P* < 0.01). This result is consistent with the previous study showing that tRNA misacylation increases upon oxidative stress [1].

Additionally, we measured the caspase-3 activity which has been proven as a cell apoptosis marker. Caspase-3 activity also increases upon CaMKII activation, and CaMKII is known to induce cell apoptosis through Caspase-3 pathway [9]. The results showed that cells triggered with Ca^2+^ stress (0.1 mM or 1 mM) had higher caspase-3 activity (*P* < 0.02) which was also confirmed by an inhibitor control (Fig 1D). Given that CaMKII protein is a multifunctional ROS sensor protein necessary for cellular Ca^2+^ homeostasis [9,10] that impacts nearly every aspect of cellular life [21], we reasoned that CaMKII protein may involve Met mistranslation for its biological activity. We therefore chose to focus on the CaMKII protein as a model system in our subsequent study.

### Identification of Met-mistranslated CaMKII proteins induced by Ca^2+^ stress

To facilitate the identification of specific Met-mistranslated CaMKII proteins, HEK293T cells were transfected with plasmids containing the wild-type, C-terminal flag-tagged CaMKIIalpha gene under the control of a constitutive promoter. Flag-tagged proteins were isolated from cells with or without Ca^2+^ stress and sent for mass spectrometry analysis (Figs 1E and S1–S6, Table 1). All screened spectra containing Met-mistranslated residues were manually inspected for mass shifts and accommodating mass spectra. Because Met-mistranslated proteins are present at low levels individually, high coverage level of the protein of interest is needed [1]. We performed the same mass spec experiment in three biological replicates and found that the number of mistranslated peptides detected generally increased with the extent of the CaMKII protein coverage (Table 1). To obtain a comprehensive number for all mistranslated peptides and a quantitative measure for the amount of mistranslated proteins would require more elaborate experiments such as SILAC or isobaric tagging together with substantially higher coverage of CaMKII. For now, our results lend qualitative support that the fraction of mistranslated peptides was greater for the Ca^2+^ stress samples.

**Table 1:** Mistranslated peptides detected in three biological replicates.

Experiment	Condition	Total # CaMKII peptides	Mistranslated	%
1	Stress	339	6 ^a^	1.8
	No stress	282	4 ^b^	1.4
2	Stress	96	5 ^c^	5.2
	No stress	106	1 ^d^	0.9
3	Stress	96	1 ^e^	1.0
	No stress	122	1 ^f^	0.8

a. Mistranslated residues: Y13M, E81M, D215M, V208M, F232M, E359M.

b. Y13M, E81M, D215M, E359M.

c. F89M, F232M, D238M, F366M (2).

d. E367M.

e. D238M.

f. D238M.

We detected a total of 9 Met-mistranslated peptides in the CaMKII protein under Ca^2+^ stress (Y13M, E81M, F89M, V208M, D215M, F232M, D238M, E359M, F366M). Five of these were also present in cells without Ca^2+^ stress (Y13M, E81M, D215M, D238M, E359M), while E367M was only detected in the no stress sample. Among these Met-mistranslated proteins detected, three are located in the association domain, while seven are located in the catalytic domain of CaMKII.

### Characterization of Met-mutant CaMKII protein activities

**Fig 2:**
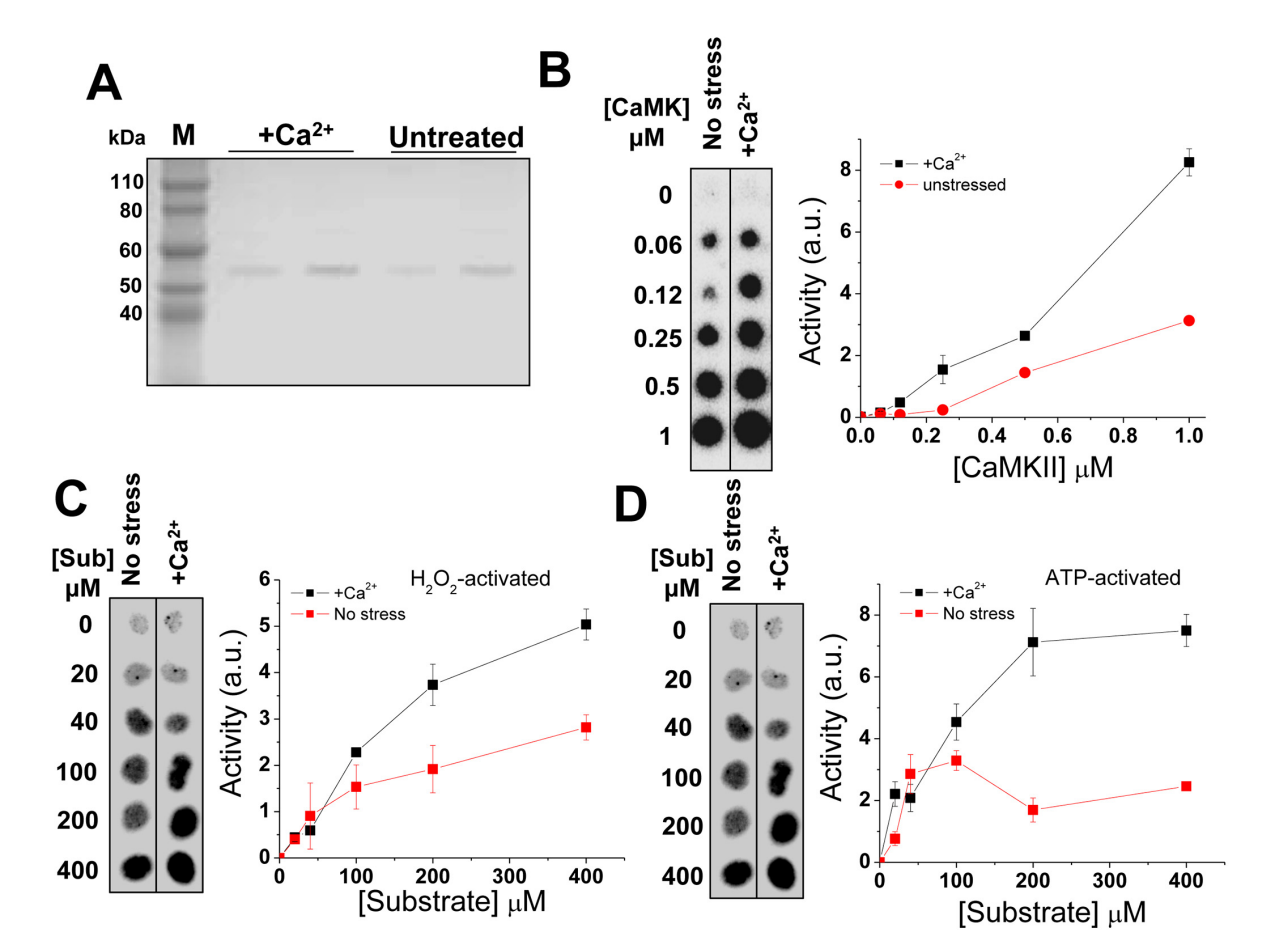
Characterization of CaMKII proteins isolated under no-stress and Ca^2+^-stress conditions. (**A**) C-terminal Flag-tagged proteins were purified from HEK293T cells and its purity examined by SDS-PAGE. Each sample is loaded in different amounts in two lanes. (**B**) Kinase activity assay of the proteins isolated from both conditions. Using the same amount of protein, CaMKII isolated under Ca^2+^ stress shows consistently higher activity than the protein isolated from no-stress condition. (**C**) Kinase activities of stressed and non-stressed samples at different substrate concentrations upon treatment with H_2_O_2_, or ATP (**D**).

We first compared the kinase activity of the purified Flag-tagged CaMKII protein samples using a well-established kinase assay [9]. The isolated CaMKII proteins were of similar purify, although the protein sample isolated from Ca^2+^ stress was expected to contain more Met-mutant proteins (Fig 2A). When the same amount of protein was used for the *in vitro* activity assay, the CaMKII sample isolated under Ca^2+^ stress showed consistently higher activity compared to those isolated in the absence of stress (Fig 2B). Kinase activities of stressed and non-stressed protein samples were also measured at different substrate concentrations, indicating that the effect of the increased kinase activity is mainly due to increased *k*_*cat*_ (Fig 2C and 2D).

CaMKII is known to be activatable in two ways, either through oxidation of the genetically encoded Met281/282 residues or through autophosphorylation of Thr286 [8,9]. Using Met281/282-oxide specific or Thr286-phospho antibodies, we found that the CaMKII protein samples obtained from the absence and presence of our Ca^2+^ stress conditions contained similar amount of oxidized or phosphorylated species (S7 Fig). We do not understand why our mild Ca^2+^ stress conditions did not trigger CaMKII activation through post-translational modifications; it may be due to the low degree of stresses used in our experiment where 0.1–1 mM of Ca^2+^ was added to the medium that initially contained 1.8 mM Ca^2+^. In any case, these results suggest that the observed differential activity per unit CaMKII protein cannot be readily explained by a greater extent of Met281/282 oxidation or higher levels of Thr286-phosphorylation, the two known mechanisms of CaMKII activation.

To directly compare the kinase activity of wild-type (WT) and Met-mutant CaMKII proteins, we generated plasmid constructs expressing WT and four mutant CaMKII proteins (E81M, V208M, F232M, and E359M). Among these mutants, two (V208M and F232M) were found under Ca^2+^ stress condition, and two (E81M and E359M) were found with and without Ca^2+^ stress. WT and mutant CaMKII proteins were expressed and purified from HEK293T cells for biochemical characterization.

**Fig 3:**
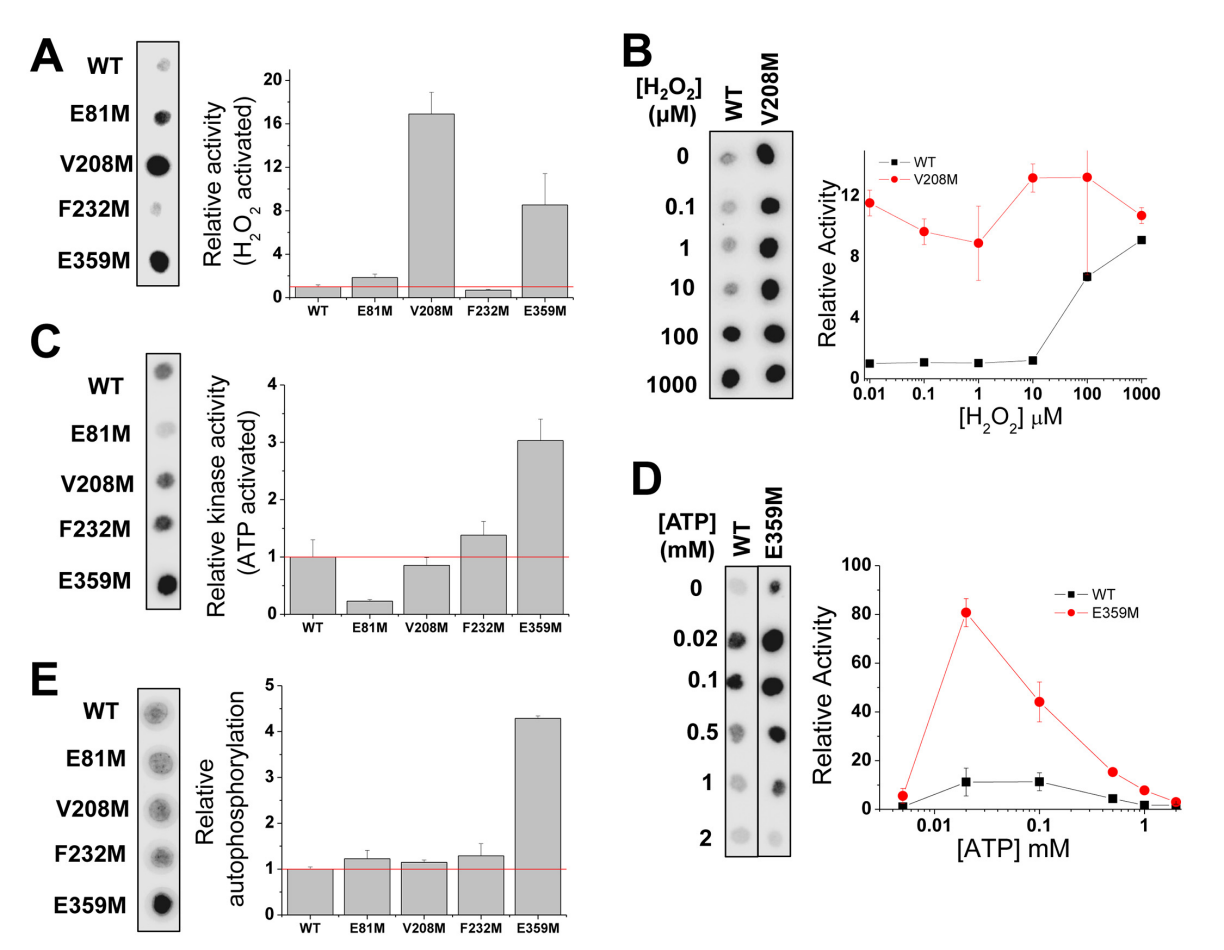
Characterization of Met-mistranslated CaMKII *in vitro*. (**A**) Comparison of kinase activity between Flag-tagged WT and four Met-mistranslated CaMKII proteins purified from HEK293T cells after treatment with 10 μM H_2_O_2_. Activities are normalized to the WT protein. (**B**) Kinase activity of the WT and V208M mutant after pre-incubation with varying H_2_O_2_ concentration (0 to 1000 μM). Activities are normalized to the WT protein at 0 H_2_O_2_ (shown at 0.01 μM in this log-plot for better visualization). (**C**) Comparison of ATP dependent kinase activity between WT and four mutant CaMKII proteins after pre-incubation with 0.1 mM ATP. Activities are normalized to the WT protein. (**D**) Kinase activity of the WT and E359M mutant after ATP pre-incubation at varying ATP concentrations (0 to 2 mM). Activities are normalized to the WT protein at 0 ATP (shown at 0.004 mM in this log-plot for better visualization). (**E**) Autophosphorylation level of WT and four mutant CaMKII proteins. Levels are normalized to the WT protein. CaMKII kinase activity was measured as a function of γ^32^P-ATP incorporation into the peptide substrate autocamtide. Autophosphorylation of CaMKII was measured as a function of γ^32^P-ATP incorporation into CaMKII protein in the absence of substrate.

We performed two well-established CaMKII activity assays using purified WT and mutant proteins in parallel *in vitro*. The first assay measures the kinase activity upon H_2_O_2_ treatment which activates CaMKII through oxidation of genetically encoded Met281/Met282 residues [9]. Using the same amount of CaMKII proteins pretreated with 10 μM H_2_O_2_, we found that 3 of the 4 mutant proteins have 2–16 fold higher activities than the WT protein (Fig 3A). We also pretreated WT and the most active V208M mutant protein with varying concentrations of H_2_O_2_ before the kinase activity assay. As expected, the WT protein became more active upon oxidation at high H_2_O_2_ concentrations, whereas the V208M mutant protein was always active at all H_2_O_2_ concentration (Fig 3B).

The second assay measures the kinase activity upon autophosphorylation of Thr286 in the presence of ATP [8]. Pretreatment with 0.1 mM ATP resulted in ~3-fold higher activity for the E359M mutant, whereas the E81M mutant has ~4-fold lower activity compared to the WT (Fig 3C). Pretreatment with varying ATP concentration (0 to 2 mM) for the WT and the E359M mutant showed that E359M had a much wider range of activation as the WT enzyme (Fig 3D). The E359M activity result correlates well with its ~4-fold higher phosphorylation levels compared to the WT CaMKII (Fig 3E).

### Subcellular levels of Met-mutant CaMKII proteins

The significantly altered biochemical properties of Met-mistranslated CaMKII proteins inspired us to explore how these mutant proteins may behave differently from the WT protein *in vivo*. We focused on the V208M and E359M mutants that showed the highest difference from the WT in our *in vitro* biochemical studies. For these three C-terminal Flag-tagged CaMKII plasmid constructs, we applied the same transfection protocol in parallel, and determined that the transfection efficiency for all three plasmids was very similar (S8 Fig).

**Fig 4:**
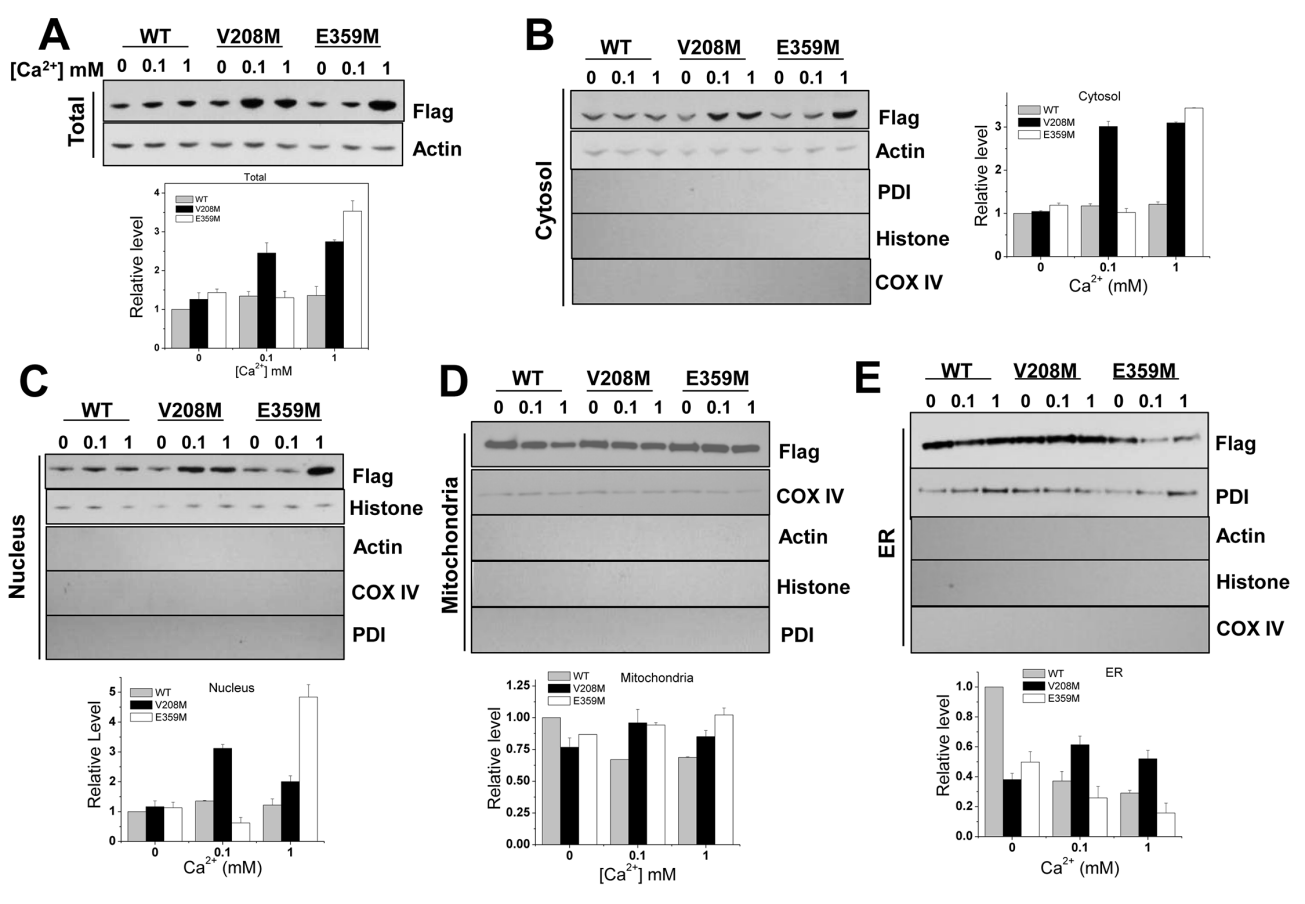
Subcellular levels of WT and two Met-mistranslated CaMKIIs. Subcelluar fractions isolated separately were subjected to SDS-PAGE followed by Western blotting to track the level of the C-terminal Flag-tagged WT and V208M, E359M CaMKII proteins. Respective subcellular markers were also shown, and control markers were used to exclude cross-contaminations. (**A**) Total lysate. (**B**) Cytoplasm. (**C**) Nucleus. (**D**) Mitochondria. (**E**) Endoplasmic reticulum. In all graphs, band signals from the CaMKII are divided by the signals from the subcellular markers, and then normalized to the WT levels at 0 mM Ca^2+^.

We examined the amount of Flag-tagged proteins in different cellular compartments by fractionation and Flag antibody Western blots together with compartment-specific cellular markers (Fig 4). The total expression levels of the WT and the two mutants were similar in the absence of stress. The expression level of both mutants increased by 2.5–4 fold when cells were exposed to 0.1–1 mM Ca^2+^, whereas the increase of the WT protein was less than 1.5-fold (Fig 4A). To compare the level of the WT and these two mutants in subcellular localizations in the absence and presence of Ca^2+^ stress, we fractionated the cell lysate into cytoplasmic, nuclear, mitochondrial and endoplasmic reticulum (ER) fractions. The cytoplasmic level reflected the differences in the total protein under all conditions (Fig 4B). The nuclear level for both mutants was generally higher than the WT protein (Fig 4C). In mitochondria (Fig 4D), the level of the WT and mutants was similar without stress; although the WT level decreased under Ca^2+^ stress, the levels for both mutants remained the same. Finally, both mutants showed lower levels than WT in the ER without stress; the WT and the E359M mutant exhibited decreased levels under Ca^2+^ stress, whereas the level of the V208M mutant remained relatively unchanged (Fig 4E). These results are consistent with our hypothesis that specific Met-mutant CaMKII proteins can exhibit distinct expression levels and subcellular localizations than the WT protein.

It is noticeable that these two mutant CaMKIIs remained at more consistent levels in mitochondria and ER upon Ca^2+^ signaling. Mitochondria and ER are major storage compartments for intracellular calcium ions [22,23]. In addition, it has been demonstrated that Ca^2+^ influx triggers translocation or redistribution of CaMKII to these subcellular compartments [14,15,16,17]. Our results suggest the possibility that the translocated or redistributed CaMKII upon Ca^2+^ influx could include disproportionally higher levels of Met-mistranslated CaMKIIs, which would enhance the functional role of the CaMKII protein.

### Met-mutant CaMKII proteins in stress-induced caspase-3 activities

**Fig 5:**
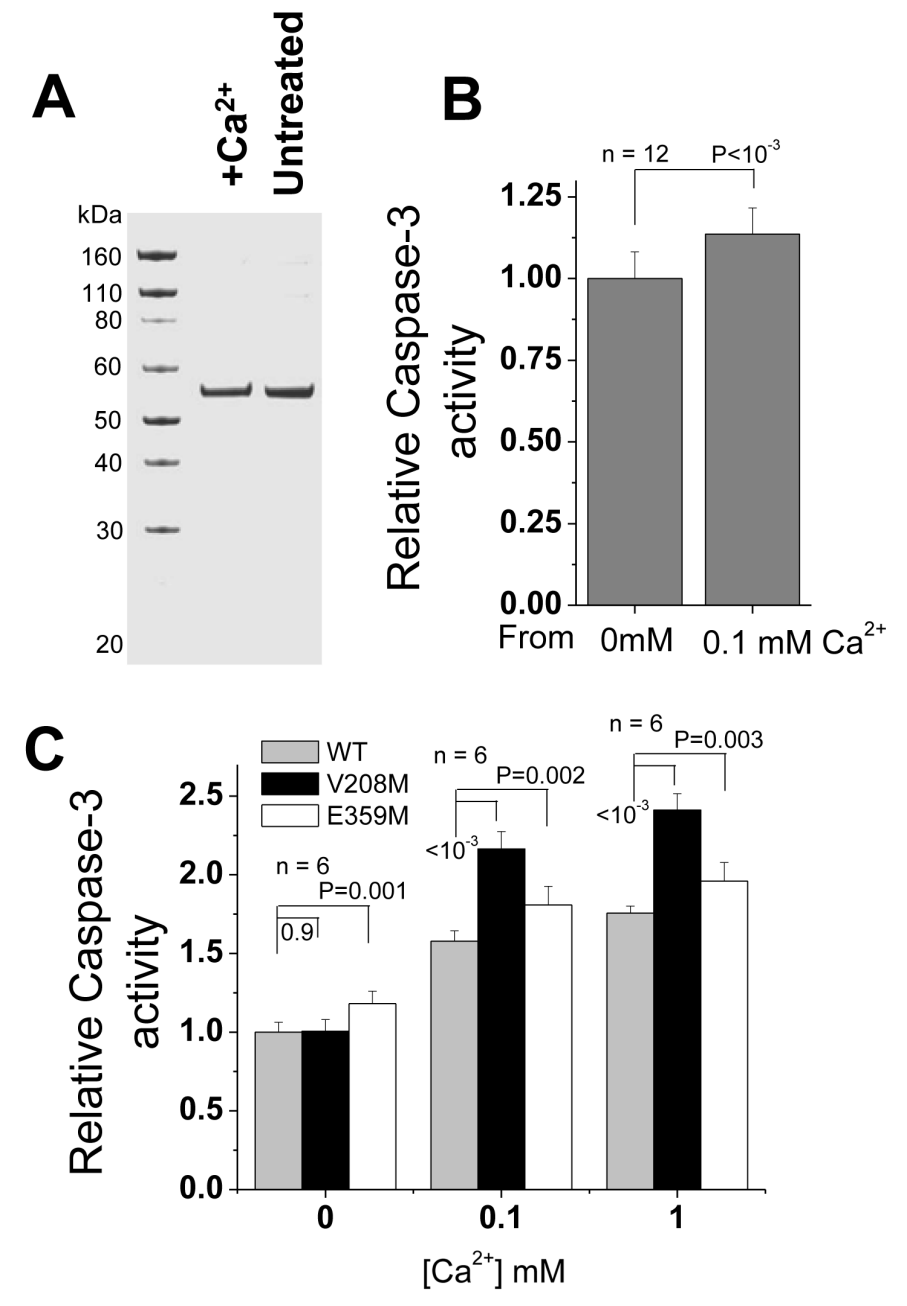
Ca^2+^ stress-induced elevation of caspase-3 activity by the WT and two Met-mistranslated CaMKIIs. **(A)** CaMKII proteins isolated under 0 and 0.1 mM Ca^2+^ conditions analyzed by SDS-PAGE. (**B**) Caspase-3 activity in cells upon transfection with CaMKII protein isolated from stressed (0.1 mM Ca^2+^) or non-stressed (0 mM Ca^2+^) conditions; n = 12 biological replicates. (**C**) Caspase-3 activity in cells upon transfection with WT or mutant CaMKIIs (V208M and E359M) plasmids, n = 6 biological replicates. In panels (B) and (C), data are expressed as Mean ± S.D. of independent replicates, data between groups are compared by Student's t test and the P values are shown.

Since excessive ROS in cells can activate CaMKII resulting in cell apoptosis [9,24], we addressed the question whether Met-mistranslated CaMKII proteins can exacerbate the effects using the caspase-3 activity assay as a proxy for apoptosis. In cells, the mistranslated CaMKII proteins are made of a mixture of at least 9 Met-substituted mutants plus the wild-type protein (Table 1). To recapitulate the biological effect and to determine whether the higher mistranslated protein mixture has inherently higher activity, we designed a protein transfection experiment to directly compare the biological activity of the protein mixture obtained from low and high mistranslation cells. We first purified CaMKII proteins from untreated, low mistranslation condition (0 mM) and 0.1 mM Ca^2+^-stressed, high mistranslation conditions through their Flag-tags (Fig 5A). When equal amounts of these two protein mixtures were directly transfected back into cells without the presence of Ca^2+^ stress, caspase-3 activity showed a marked increase when the protein from the high mistranslation condition was used (Fig 5B, *P* < 0.01). This result validates the higher biological activity of the protein mixture from Ca^2+^-stressed cells as demonstrated *in vitro* in Fig 2B.

We also transfected the WT and V208M/E359M plasmids and measured the effect of overexpressing WT and the specific Met-substituted mutants on caspase-3 activity. The CaMKII proteins generated in this experiment do not correspond to the actual mixture of mistranslated proteins; rather, this experiment is designed to show the effect of individual CaMKII mutants present under mistranslation conditions. Since HEK293T cells also contain substantial amount of endogenous CaMKII proteins (S7 Fig), the observed caspase-3 activity in our experiment corresponds to the cumulative effects of the endogenous and the transfected CaMKII WT and mutant proteins. Caspase-3 activity assay shows that both mutant proteins indeed have higher *in vivo* activity as the WT CaMKII (Fig 5C), especially when cells were treated with 0.1 or 1 mM Ca^2+^ (*P* < 0.01). The increase for the V208M is ~1.4-fold and for the E359M is ~1.2-fold. E359M also shows a higher basal level without stress. Since the endogenous protein mixtures are always present in the same amount and composition, the effect of the transfected mutant proteins over the transfected wild-type protein represents a lower limit of their biological activity. These results indicate that the Met-mistranslated mutant proteins can have significantly enhanced physiological effects compared to the WT protein.

We also measured the cell apoptotic effects of WT, V208M and E359M mutants using the TUNEL assay (S9 Fig). Without Ca^2+^ stress, the basal cell apoptosis level was 5–6% for WT and V208M mutant, but ~15% for the E359M mutant. In the presence of 0.1 mM Ca^2+^, cell apoptosis level increase for the WT was ~1.3-fold, but ~3-fold for the V208M mutant; whereas the E359M mutant showed a reduction of ~1.4-fold. For both V208M and E359M mutants, however, the apoptosis levels were consistently higher at 0.1–1 mM Ca^2+^ than the WT protein. Although the caspase-3 and the TUNEL assays did not show the same trend upon Ca^2+^ stress, the observed difference between the assays may be derived from subjective choice of areas counted under the microscope in the TUNEL assay or from real cell culturing effects. The TUNEL assay used cells cultured on microscope slides, whereas the caspase-3 assay used cells cultured in 96-well plates followed by lysis. For quantitative purposes, caspase-3 assay homogenizes cell lysate for the entire well and is better than the TUNEL assay which is confined to counting in several areas under the microscope’s field of view. In any case, both caspase-3 and TUNEL results are largely in accord with the expression level analysis, and indicate that these two Met-mistranslated mutant proteins can have significantly enhanced physiological effects compared to the WT protein.

The observed caspase-3 activity of the V208M and E359M mutants seems to be consistent with their cellular occurrence as determined by our mass spectrometry analysis (Table 1). The E359M mutant was already present in the absence of Ca^2+^ stress, and this mutant induced higher caspase-3 activity under no stress condition. The V208M mutant was only present in the presence of Ca^2+^ stress, and this mutant induced higher aspase-3 activity only under Ca^2+^ stress.

## Discussion

A central pillar of molecular biology is the accuracy of information transfer from DNA to protein. Overall translational fidelity is critical for cell survival and it is commonly believed that the genetic code should be obeyed during protein synthesis. However, accumulating evidence suggests that deliberate synthesis of mutant proteins can have biological roles in cellular stress response, and mistranslation may represent a unique form of environmental adaptation [3,25]. For example, mycobacteria could obtain phenotypic resistance to antibiotics by altering translational fidelity [4]; human fungal pathogen *Candida albicans* could generate cell surface variability through CUG mistranslation [26]. Mycoplasma parasites could employ mistranslation as a mechanism for antigen diversity to escape host defense [27]. Our discovery that mammalian cells can regulate Met-mutant protein production through tRNA misacylation has been shown to play a beneficial role through enhanced response to oxidative stress [1,6]. Specific bacterial protein mixtures under mistranslation conditions have been isolated and shown to have distinct activity from the wild-type protein *in vitro* [4,28]. To our knowledge, however, it was not known whether any specific, mistranslated protein molecule exhibits distinct biochemical and biological properties as the genetically encoded, WT protein. We have now addressed this question in our *in vitro* and *in vivo* analysis of specific, Met-mistranslation generated mutant proteins and shown that such mutants can have distinct properties as the WT protein.

**Fig 6:**
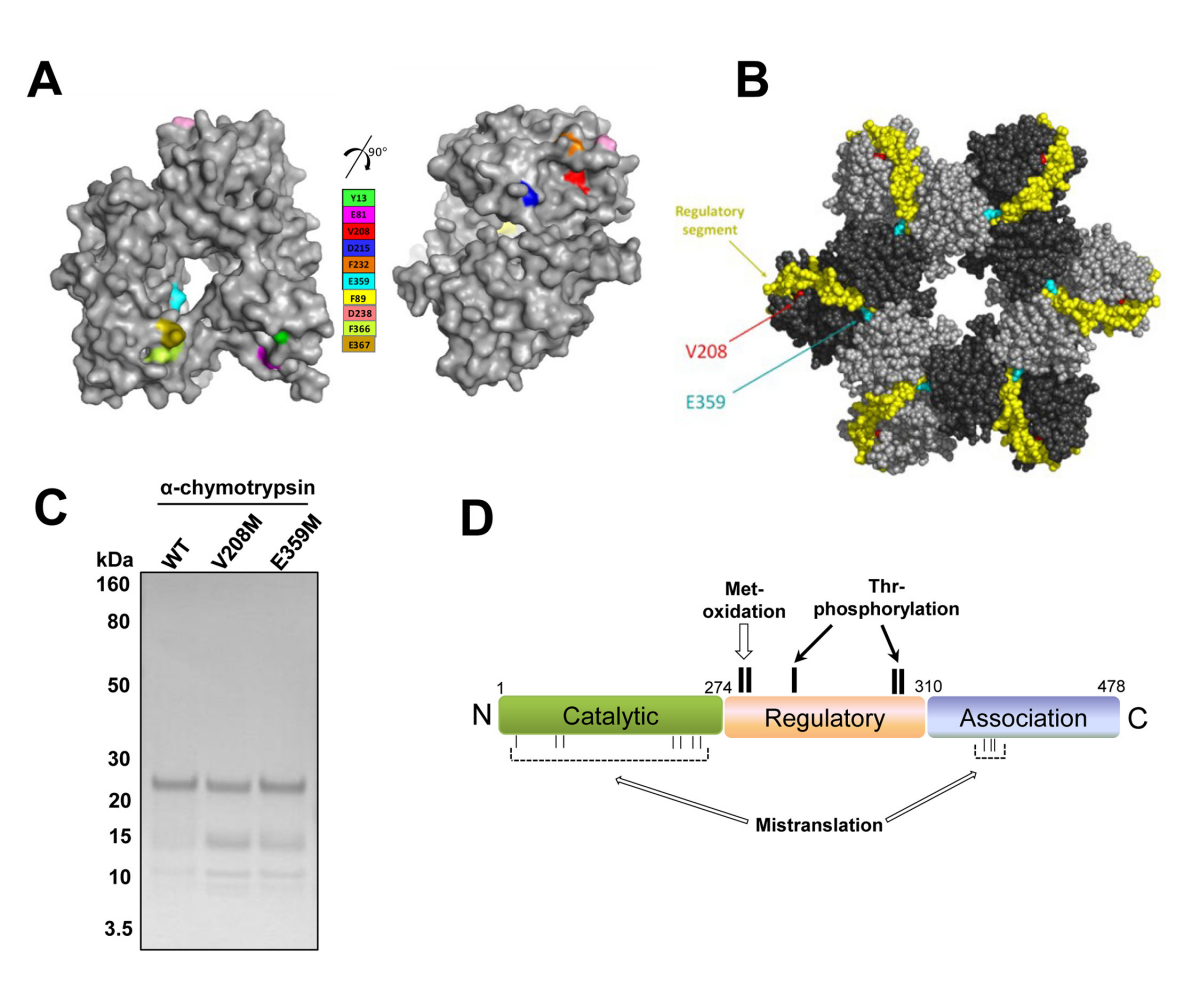
Met-mistranslation as nature’s way to expand the activity profile of proteomes. (**A**) The location of the 10 Met-mistranslated CaMKII residues detected in this work in the monomeric structure of CaMKII (PDB file 3SOA). Met-mistranslated substitutions are generally located on the surface of the CaMKII monomer. (**B**) The location of the V208M (red) and E359M (cyan) mutants in the hexamer ring of CaMKII (3SOA). The Ca^2+^/calmodulin regulatory segment is colored in dark yellow. V208 is at the base of the regulatory segment, and E359 is at the interface between subunits. (**C**) Limited proteolysis of WT and V208M/E359M mutant CaMKII proteins visualized using Coomassie staining. (**D**) A functional model of Met mistranslation on CaMKII activity. Known post-translational modifications that activate CaMKII include Met281/282 oxidation and Thr287/Thr306/Thr307 phosphorylation (above the domain structure of CaMKII). Mistranslation (below the domain structure) generates many mutant proteins, individually at low frequency, but collectively affecting CaMKII activity.

Our model system here is the CaMKII protein kinase, a crucial component in the cellular Ca^2+^ signaling network [29]. Calcium is essential for life and impacts cell physiology in many ways [21]. We were first inspired by our finding that Ca^2+^ stress also triggered higher level of tRNA misacylation in a mammalian cell. We therefore investigated whether Ca^2+^ stress-mediated Met-mistranslation can have biological consequences on a specific protein involved in Ca^2+^ signaling. Previous evidence that CaMKII could be activated through autophosphorylation upon Ca^2+^/calmodulin binding or through oxidation of generally encoded methionine by ROS [9,30] motivated us to select CaMKII as our protein of interest. We identified a total of 10 specific Met-mistranslated CaMKII proteins from cells. CaMKII holoenzyme is a dodecamer made of two hexameric rings [31]. These mistranslated Met-residues are located on the surface of the monomeric CaMKII protein (Fig 6A). This preferred surface location of the Met-mutations in CaMKII is similar to a previous study that mapped the location of Met-mistranslated residues in another human protein, AIMP3 [6].

At the hexameric level, however, the two specific mutations V208M and E359M studied extensively in this work are located at places crucial to the catalytic activity of CaMKII (Fig 6B). E359 is located at the subunit interface in the hexameric ring. Hence, the effects observed for the E359M mutant may be due to the alteration of subunit interactions in the catalytic cycle. V208 is located at the base of the regulatory segment that undergoes a very large conformational change upon Ca^2+^/calmodulin binding [31]. The effects observed for the V208M mutant may be due to its effect on this conformational change crucial for CaMKII catalysis. To evaluate the conformational differences between the WT and these two mutant proteins, we performed limited proteolysis of purified CaMKII proteins. Using α-chymotrypsin, we identified distinctly stable protein fragments for both mutant proteins, demonstrating that these mutants indeed have distinct conformations as the WT protein (Fig 6C). The exact molecular mechanism on why these specific Met-mistranslated mutants achieve their distinct activities from the WT protein requires further investigation.

CaMKII is known to be activatable either through oxidation of the genetically encoded Met281/282 residues or through autophosphorylation of Thr286 [8,9]. In this study, we found that the CaMKII proteins obtained from the absence and presence of Ca^2+^ stress contained similar amount of Met281/282 oxidized or Thr286-phosphorylated species (S7 Fig), hence the observed differential activity is more consistent with contributions from mistranslation. A fundamental difference between post-translational modification versus mistranslation on protein activity is that post-translational modifications are typically confined within one or just a handful of residues at high frequency, whereas mistranslation can produce a large number of protein mutants at low frequency individually. Although the mistranslated proteins may contribute less individually, they can have an appreciable effect collectively (Fig 6D). Our result is consistent with Met mistranslation being able to operate under mild stress conditions where post-translational modifications may not be triggered.

Under Ca^2+^ stress, the subcellular localization of the V208M/E359M mutant CaMKII proteins shows quantitative differences from the WT protein; it is notable that a mutant CaMKII can be present at a higher level in mitochondria or endoplasmic reticulum under Ca^2+^ stress conditions. Mitochondria and ER are major storage compartments for intracellular calcium ions [22,23]. Our results are consistent with enhanced translocation and/or stability of Met-mistranslated CaMKII proteins to these compartments upon Ca^2+^ stress, in order to better utilize the properties of Met-mistranslated proteins.

Consistent with previous studies, Met-mistranslated CaMKIIs could be identified in cells under normal and stress conditions, and two among the four mutant proteins we studied were either of similar activity or less active than the WT protein. These other Met-mutant CaMKIIs could be used for other, unknown pathways. Additionally, Met-mistranslated but misfolded proteins may also be scavenged through degradation pathways [3,32,33]. We do not know at this time how the Met-misacylated tRNAs are selectively used by the elongation factors and the ribosome. But, it is conceivable that these misacylated tRNAs may not be used equally at all mRNA codons due to selective interaction with the elongation factors or with the ribosome [34,35]. Selective use of Met-misacylated tRNAs may increase the amount of specific Met-mistranslated proteins beyond the respective level of misacylated tRNAs.

In summary, this work demonstrates that naturally occurring Met-mistranslation can generate specific mutant proteins with different properties than the wild-type protein. Met-mistranslation broadens the activity profile of proteins from the same gene that are otherwise confined by its genetic code (S10 Fig). When translation is highly accurate, all protein molecules synthesized from the same gene are chemically identical, its activity distribution is limited to differences in folding and dynamics of individual molecules. Under conditions of Met-mistranslation, chemical diversity is introduced for protein molecules synthesized from the same gene. This chemical diversity can expand the activity profile, resulting in mutant proteins such as the V208M and E359M CaMKII that are significantly more active than the wild-type protein. Met-mistranslation is a nature’s way to bypasses the protein sequence constrained by the genetic code in order to generate protein variants that can be beneficial for stress response and adaptation.

## Methods

### Methionine misacylation under Ca^2+^ stress

tRNA misacylation was measured using a microarray method described previously [1]. Briefly, human embryonic kidney 293T (HEK293T) cells were cultured in DMEM (Invitrogen, Carlsbad, CA) supplemented with 10% FBS (HyClone) at 37°C with 5% CO_2_. Cells were grown on 10 cm plates to ~80% confluency. For pulse-labeling experiments, 0.5 mCi [^35^S]-Met (Perkin-Elmer, Boston, MA) was added onto each plate and incubated at 37°C for 8 min. Cells were washed twice with 300 mM NaOAc/HOAc pH4.8/10 mM EDTA and then scraped from the plate. Cells were transferred to tubes and spun down. Pellets were resuspended in ice-cold 300 mM NaOAc/HOAc pH4.8/10 mM EDTA. Charged tRNAs were isolated using acetate-saturated phenol/CHCl_3_ (pH 4.8). RNAs were precipitated using equal volume of isopropanol and finally dissolved in 10 mM NaOAc/HOAc pH4.8/1mM EDTA. RNA concentration was measured using Nanodrop 2000 (Thermo Scientific) and 20 μg RNA was used for array hybridization.

### Expression and purification of Met-mistranslated CaMKII proteins from mammalian cells

HEK293T cells were transfected with 24 μg flag-tagged DNA encoding CaMKII alpha (GenBank Accession No. AAH40457.1, OriGene Technologies) using Lipofectamine 2000 (Invitrogen) and incubated for 6 h. Culture medium was changed to fresh medium with or without 0.1 mM CaCl_2_ and cells were incubated for 16 h. Cells were lysed in mammalian cell lysis CelLyticM (Sigma) including fresh Protease Inhibitor Cocktails. Cell lysates were centrifuged and the supernatant was incubated with ANTI-FLAG M2 Affinity Gel (Sigma) overnight at 4°C. Flag-tagged CaMKII was eluted with FLAG peptide (Sigma) after stringent washes. The FLAG peptide was removed by Amicon filter of 10-kDa (Millipore). Protein concentration was measured by Quick Start Bradford (Bio-Rad) and protein purity was checked by SDS-PAGE with Coomassie staining.

### Identification of Met-substituted residues in CaMKII protein using mass spectrometry

For mass spectrometry, 20 μg flag-tagged CaMKII purified from 0.1 mM Ca^2+^ stress and control conditions was sent to the Proteomics and Mass Spectrometry Facility at Donald Danforth Plant Science Center (St. Louis, MO). The protein was digested with trypsin at 37°C overnight and the LC-MS/MS was carried out on a LTQ-Orbitrap Velos Pro (ThermoFisher Scientific, Waltham, MA) coupled with a U3000 RSLCnano HPLC (ThermoFisher Scientific, Waltham, MA). The raw data were analyzed using Progenesis-QI (Nonlinear dynamics). The chromatograms were aligned and the MS/MS data was extracted for peptide identification using Mascot (Matrix Science, London, UK; version 2.5.1). FASTA sequences of CaMKII were created by substituting other 19 amino acids to methionine residue. Mascot [36] and MaxQuant [37] software were used to identify peptides containing Met substitutions. Mascot was searched with a fragment ion mass tolerance of 0.80 Da and a parent ion tolerance of 15 PPM assuming the digestion enzyme trypsin. Carbamidomethylation of cysteine was specified in Mascot as fixed modification. Deamidated of asparagine and glutamine and oxidation of methionine were specified as variable modifications. All spectra containing Met-mistranslated residues with mass shift were manually inspected. The identified Met-mistranslated residues were mapped to the structure of CaMKII alpha (3SOA) and visualized using PyMOL software.

The level of Met mistranslation at each codon position can depend on the levels of mismethionylated tRNAs for the codon and the utilization of the mismethionylated tRNAs by the ribosome and translation factors. Although the level of tRNA mismethionylation can be semi-quantitatively measured by microarrays, currently we do not know how these tRNAs are used in subsequent steps in translation. Individual tRNAs can be misacylated up to ~1% of the charged, cognate tRNA^Met^s [1]. To identify Met-mistranslated peptides at such levels, very high coverage of the specific protein is required. We repeated the mass spec experiment (Fig 1) three times, each time obtaining different levels of coverage for the wild-type CaMKII protein. The data from highest level of coverage is shown in Fig 1. When the total peptide coverage is reduced, the number of detectable mistranslated peptides also decreased. However, identical, mistranslated peptides can still be found (S5 Fig).

### Mutagenesis and purification of WT and MT CaMKII from mammalian cells

Four mutations (E81M, V208M, F232M and E359M) from mass spectrometry analysis were chosen for further experiments using the QuikChange Site Directed Mutagenesis method (Stratagene). The oligonucleotide primers were designed according to Agilent Technologies. Generally, PCR product of each mutant was amplified by *PfuTurbo* DNA polymerase using WT flag-tagged CaMKII plasmid as the template. PCR products were then digested with *Dpn I* (New England Biolabs) to remove template DNA followed by transformation into XL1-Blue supercompetent cells (Agilent Technologies). Mutant flag-tagged CaMKII plasmids extracted from individual colonies were confirmed by Sanger sequencing. WT and mutant CaMKII proteins were expressed and purified from HEK293T cells as described under mass spectrometry experiments.

### Kinase activity of WT and MT CaMKIIs *in vitro*

CaMKII kinase activity assays were performed according to previous studies with minor modifications [9]. These assays have been used extensively in the field. To compare kinase activity between WT and mutant CaMKIIs, we measured kinase activity of CaMKII in the presence of H_2_O_2_ or ATP, which are activators of CaMKII. For H_2_O_2_ or ATP dependent kinase activity, 0.4 μM purified CaMKII protein was pretreated with 1 mM CaCl_2_/2 μM calmodulin (Millipore) in reaction buffer (50 mM TrisHCl pH7.5, 10 mM MgCl_2_, 0.5 mM DTT) on ice for 10 min. The protein was then exposed to H_2_O_2_ or ATP at indicated concentrations for 30 min at 37°C. 10 mM EGTA were added and the samples were incubated for another 30 min at 37°C. 100 μM synthetic peptide substrate autocamtide 2 (Millipore) containing [γ-^32^P] ATP were added to the enzyme mixture and the reactions proceeded for 30 min at 37°C. The reaction mixture was spotted onto P81 paper (Whatman) followed by stringent washes with 0.75% phosphoric acid. The P81 papers were exposed to phosphorimagering (BioRad) and quantified using the phosphorimager software. Kinase activities of Met-mistranslated CaMKII proteins were also profiled under different substrate concentrations (0–400 μM).

### Autophosphorylation of CaMKII

To measure autophosphorylation level of CaMKII, purified protein was pretreated with 1 mM CaCl_2_/2 μM calmodulin in reaction buffer on ice for 10 min. Samples were then treated with 10 mM EGTA for 30 min at 37°C to remove Ca^2+^. 100 μM cold ATP containing [γ-^32^P] ATP was added and the reaction proceeded for 30 min at 37°C. CaMKII autophosphorylation level was measured as a function of ^32^P-ATP incorporation into CaMKII protein.

### Preparation of cellular fractions overexpressing Met-mistranslated CaMKII

Flag-tagged WT and MT CaMKII DNAs were transfected into HEK293T cells using Lipofectamine 2000 as described above. Culture medium was changed to fresh medium containing various concentration of Ca^2+^ (0 mM, 0.1 mM and 1 mM) and cells were incubated for 16 h. Cytoplasmic and nuclear fractions were extracted separately using NE-PER Nuclear and Cytoplasmic Extraction Reagents (Thermo Scientific) according to the instructions of manufacturers. We further isolated mitochondria or endoplasmic reticulum fractions considering their role of calcium metabolism [24]. Mitochondria and endoplasmic reticulum fractions were separated from cells using Mitochondria Isolation Kit (Thermo Scientific) and Endoplasmic Reticulum Isolation Kit (Sigma), respectively. Protein concentrations were adjusted for Western Blotting analysis.

### Western blotting

Total lysate or cytoplasmic or nuclear or mitochondria or endoplasmic reticulum fractions were subjected to SDS-PAGE followed by transferring to PVDF membrane (Millipore). PVDF membranes with different fractions were incubated with mouse anti-flag antibody (1:5,000 in TBST, Origene) at RT for 2 h. Rabbit anti-actin antibody (1:5,000, Sigma), rabbit anti-histone H2A.Z antibody (1:5,000 in TBST, Cell signaling Technology), rabbit monoclonal anti-COX IV antibody (1:5,000, Mitochondrial Loading Control, Abcam) and rabbit monoclonal anti-PDI antibody (1:5,000, Cell signaling Technology) were used as loading control for cytoplasm, nucleus, mitochondria, and endoplasmic reticulum, respectively. Control antibodies were used to exclude cross-contaminations between cellular fractions. After washing, PVDF membranes were incubated with anti-mouse HRP conjugated IgG (1:10,000) and anti-rabbit HRP conjugated IgG (1:10,000) at RT for 30 min. Immune blot bands were visualized by ECL method and quantified using Image J Software (NIH).

### Cellular ROS measurements

HEK293T cells were seeded in 96-well cell culture plates, cells were cultured in complete medium without phenol red (Gibco, life technologies) at 37°C with 5% CO_2_. At ~80% confluency (at least 48 h after seeding), Ca^2+^ was added at different concentrations (0, 0.1 or 1 mM) and cells were incubated for another 16 h. Cellular ROS level was measured using DCFDA Cellular ROS Detection Assay Kit (Abcam) according to manufacturer's instruction. 2’,7’-dichlorofluorescin diacetate (DCFDA) was added to cells for 1 h and fluorescent intensity was obtained at 485 nm/535 nm. Tert-butyl hydrogen peroxide (TBHP) was used as positive control, and blank wells with substrate DCFDA were used as negative controls.

### Caspase-3 activity measurements

#### Caspase-3 assays

HEK293T cells were seeded in 6-well cell culture plates. At ~80% confluency, CaCl_2_ were added to culture medium (0 mM, 0.1 mM, 1 mM) and cells were incubated for 16 h. Caspase-3 activity, a cell apoptosis marker [9,38,39] was determined using EnzChek Caspase-3 Kit (Molecular Probes) according to manufacturer's instruction. Z-DEVD-AMC was used as the substrate and Ac-DEVD-CHO was used as the inhibitor to confirm caspase-3 activity. Fluorescent intensity was obtained at 342 nm/441 nm.

#### CaMKII protein transfection

To demonstrate that the CaMKII proteins from the low and high mistranslating cells have distinct physiological effects in cells, we transfected the purified protein mixtures isolated from stressed (0.1 mM CaCl_2_) or non-stressed (0 mM CaCl_2_) conditions back to cells. HEK293T cells were seeded in 6-well cell culture plates, at ~80% confluency, 5 µg of proteins were transfected into cells using Xfect Protein Transfection Reagent (TaKaRa/Clontech). Cells were incubated with transfection reagent/protein mixture for 1 h and caspase-3 activity was subsequently measured as above. The concentration of the protein mixtures used in transfection was measured first by A_280_ absorbance, then run on SDS-PAGE to ensure purify and same quantity (Fig 5A).

The Xfect Protein Transfection method uses a modified peptide with cell-penetrating activity whose amino acid sequence enables it to interact with the protein cargo and transports the protein across cell membrane. According to manufacturer’s cellular transfection data, transfected proteins through this method can be present in the cytosol, mitochondria and nucleus.

#### Plasmid transfection

HEK293T cells were seeded in 6-well cell culture plates, cells at ~80% confluency were transfected with empty Flag-pCMV5, Flag-WT or mutant CaMKII plasmids using Lipofectamine 2000. 6 h post transfection, culture medium was changed to fresh medium containing 0, 0.1 or 1 mM Ca^2+^ and further incubated for 16 h. Caspase-3 activity was measured the next day as described above.

### Cell apoptosis

Cell apoptosis level was measured by TUNEL assays using In Situ Cell Death Detection Kit (Roche) according to previous reports [9,24]. Fluorescent images were taken under confocal microscopy and analyzed using Image J software.

### Limited proteolysis of Met-mistranslated CaMKII proteins

Limited proteolysis assay was carried out according to previous reports [40,41]. Briefly, in a 25-μl mixture, 16 μg of CaMKII proteins (WT, V208M and E359M) was incubated with 500 ng of α-chymotrypsin (1/32, w/w) at 25°C for 30 min. The proteolytic digestion was terminated by adding SDS sample buffer X 4 (with 5.2 mM PMSF & 5.2 mM EDTA), samples were boiled at 100°C for 5 min. Digested fractions were analyzed by SDS-PAGE using Coomassie staining.

### Statistical analysis

Data in all assays are expressed as Mean ± S.D. of independent replicates, and data between groups were compared by Student's t test. The *P* value of <0.05 was considered to be a significant difference.

## Supporting Information

S1 Fig Mass spec identification of the Y13M peptide in CaMKII. (TIF)

S2 Fig Mass spec identification of the E81M peptide in CaMKII. (TIF)

S3 Fig Mass spec identification of the V208M peptide in CaMKII. (TIF)

S4 Fig Mass spec identification of the D215M peptide in CaMKII. (TIF)

S5 Fig Mass spec identification of the F232M peptide in CaMKII. Spectra are shown from two independent experiments.
(TIF)

S6 Fig Mass spec identification of the E359M peptide in CaMKII. (TIF)

S7 Fig Additional characterization of CaMKII proteins isolated under no-stress and Ca^2+^-stress conditions. (A) In cell lysate, the level of Met281/282 oxidation was similar under no-stress or Ca^2+^ stress conditions. (B) In cell lysate, the level of Thr286-phosphorylation was also similar under no-stress or Ca^2+^ stress conditions.
(TIF)

S8 Fig Transfection efficiency of the WT and V208M, E359M plasmids. (TIF)

S9 Fig Ca^2+^ stress-induced cell apoptosis by the WT and two Met-mistranslated CaMKIIs by TUNEL assay. (A) Cells expressing WT or mutant CaMKIIs (V208M and E359M) were treated without or with 0.1 and 1 mM Ca^2+^. Cell apoptosis level was measured by TUNEL assays. Fluorescent images were taken using confocal microscopy under 10X objective. Scale bars equal 50 μm. (B) Quantitation of the TUNEL assay results. Six images views were taken for each condition and more than 1,000 cells were used for apoptosis level quantification by calculating the ratio of TUNEL/DAPI.
(TIF)

S10 Fig Met-mistranslation expands the activity profile of proteins derived from the same gene. (TIF)
