# Correction: Methionine Mistranslation Bypasses the Restraint of the Genetic Code to Generate Mutant Proteins with Distinct Activities

**DOI:** 10.1371/journal.pgen.1005832

**Published:** 2016-01-25

**Authors:** Xiaoyun Wang, Tao Pan

Fig 2 is incorrect. The images in panels C and D are duplicated. The authors have provided a corrected version of Fig 2 here.

**Fig 2:**
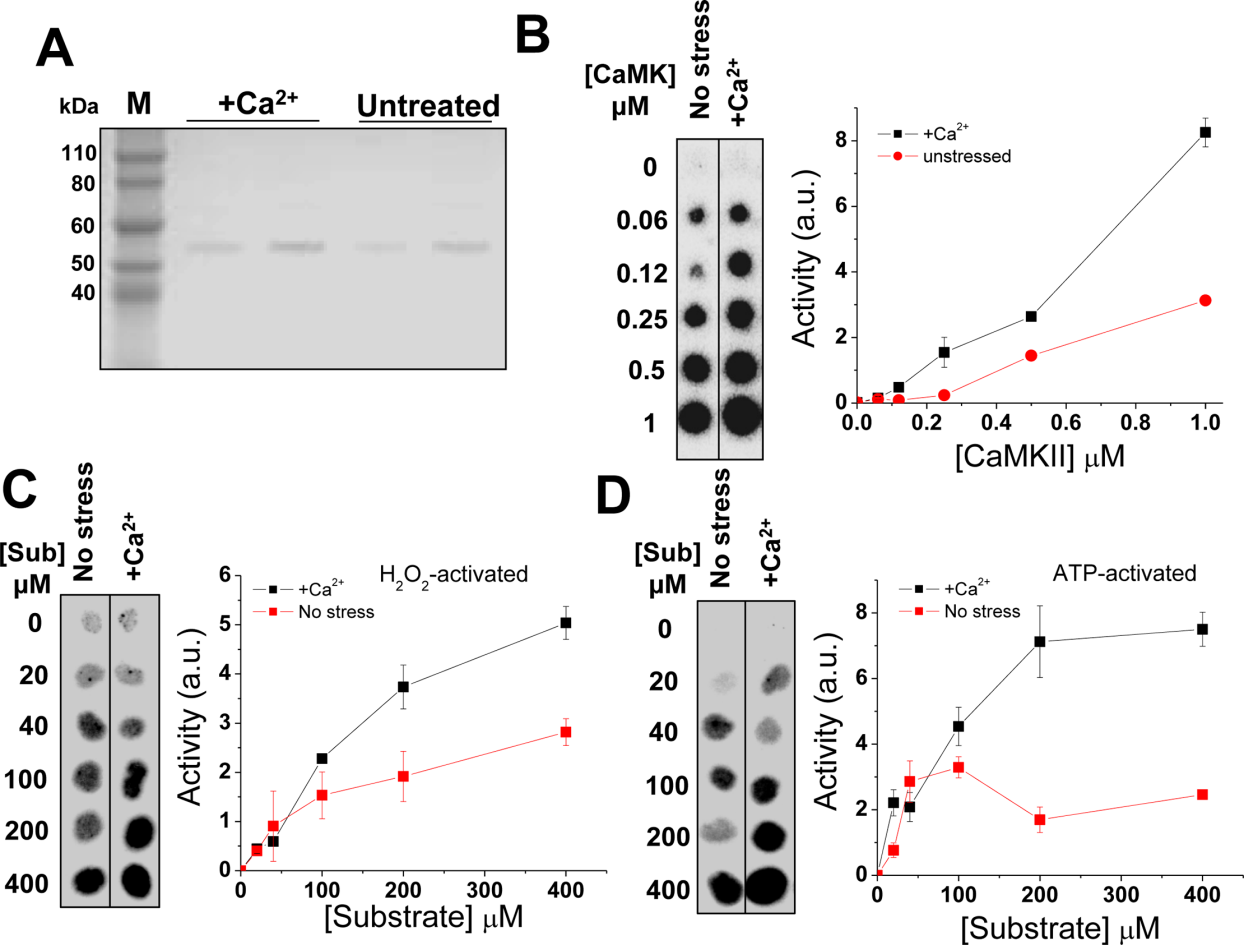
Characterization of CaMKII proteins isolated under no-stress and Ca^2+^-stress conditions. **(A)** C-terminal Flag-tagged proteins were purified from HEK293T cells and its purity examined by SDS-PAGE. Each sample is loaded in different amounts in two lanes. **(B)** Kinase activity assay of the proteins isolated from both conditions. Using the same amount of protein, CaMKII isolated under Ca^2+^ stress shows consistently higher activity than the protein isolated from no-stress condition. **(C)** Kinase activities of stressed and non-stressed samples at different substrate concentrations upon treatment with H_2_O_2_, or ATP **(D)**.
