# Supplementary figures and images for: Methionine Mistranslation Bypasses the Restraint of the Genetic Code to Generate Mutant Proteins with Distinct Activities
Source: PLoS Genet. 2015 Dec 28;11(12):e1005745. doi: 10.1371/journal.pgen.1005745 (PMC4692448; doi:10.1371/journal.pgen.1005745)

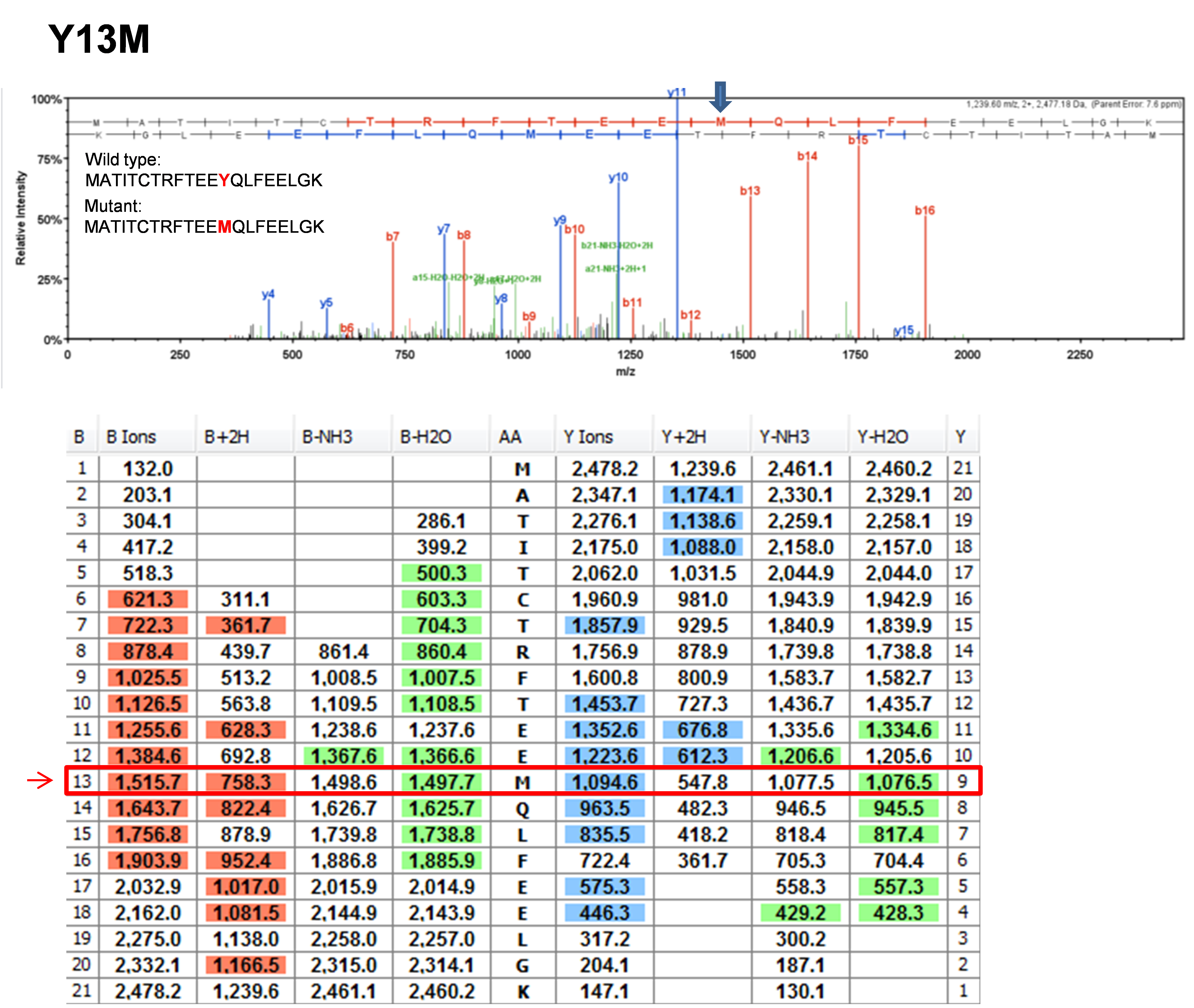

Supplement: S1 Fig — (TIF) [file pgen.1005745.s001.tif]

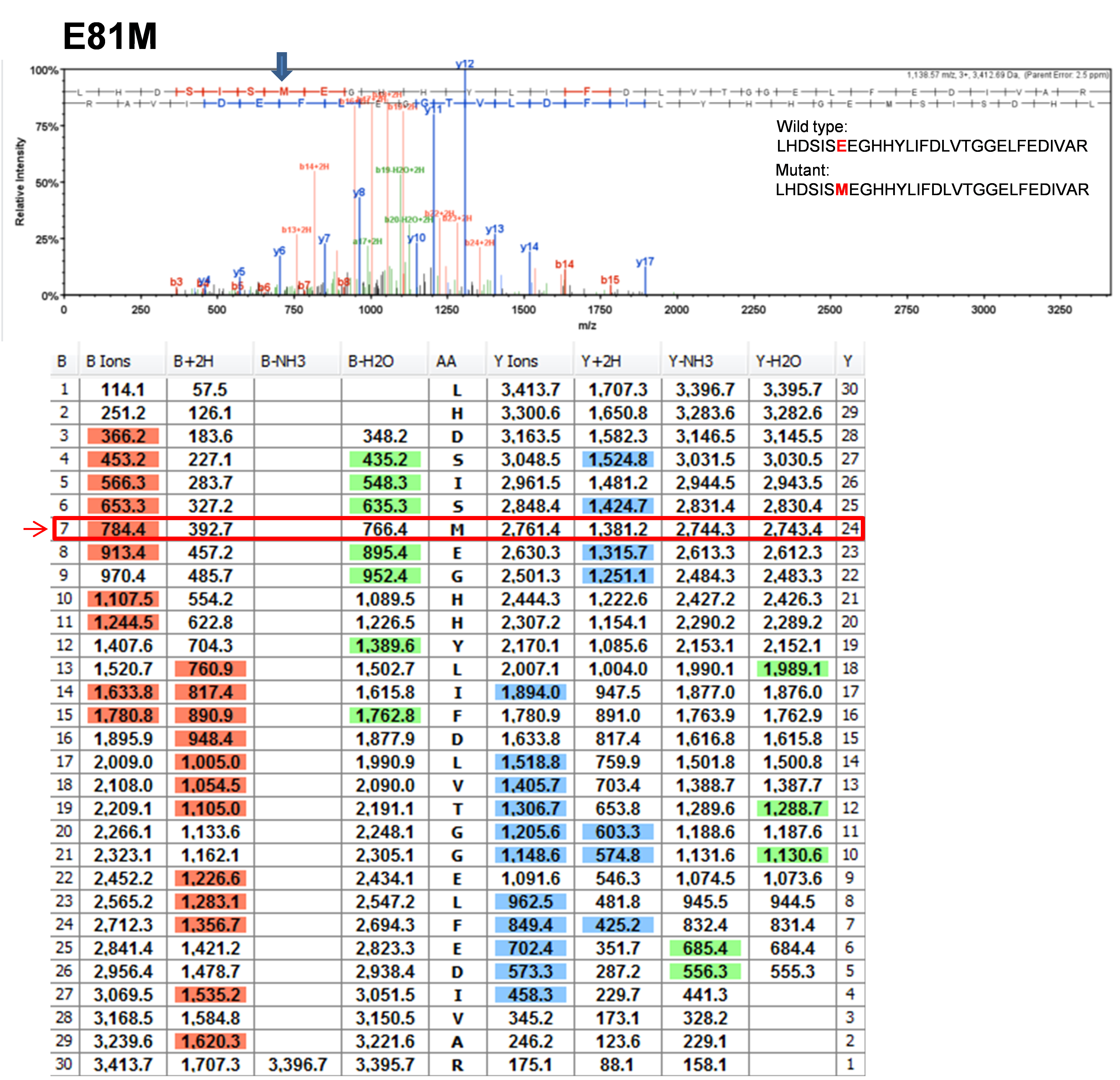

Supplement: S2 Fig — (TIF) [file pgen.1005745.s002.tif]

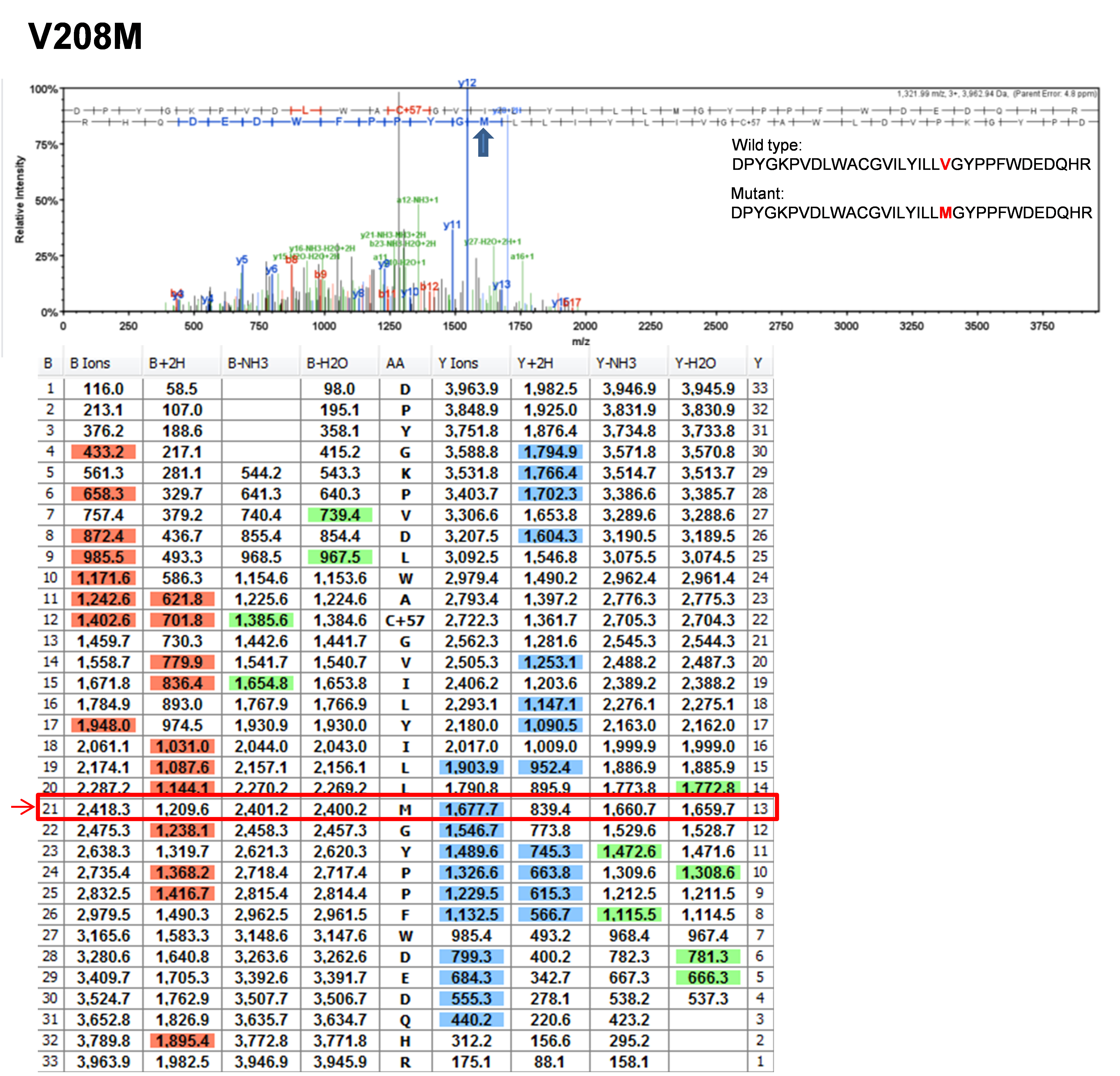

Supplement: S3 Fig — (TIF) [file pgen.1005745.s003.tif]

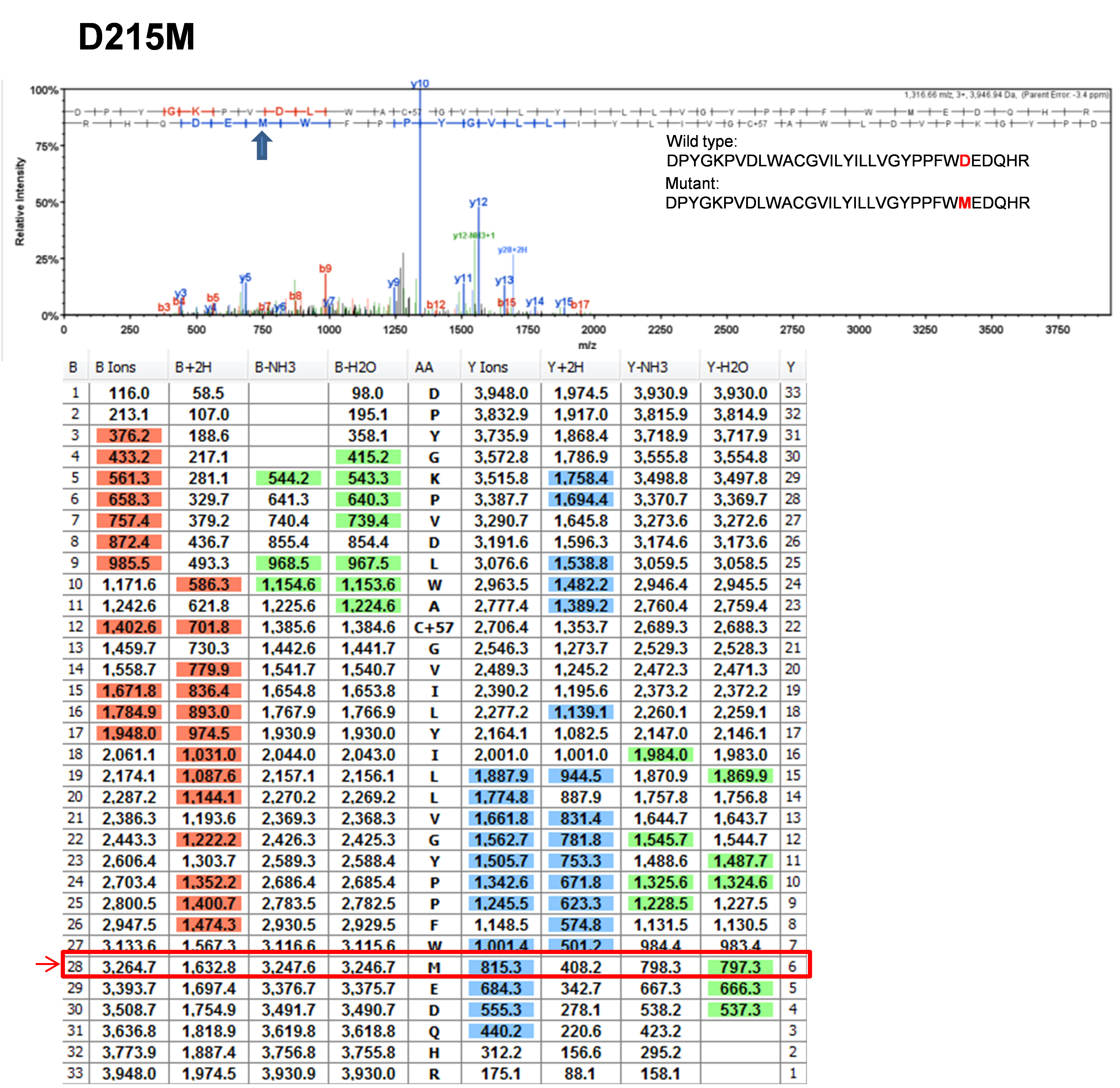

Supplement: S4 Fig — (TIF) [file pgen.1005745.s004.tif]

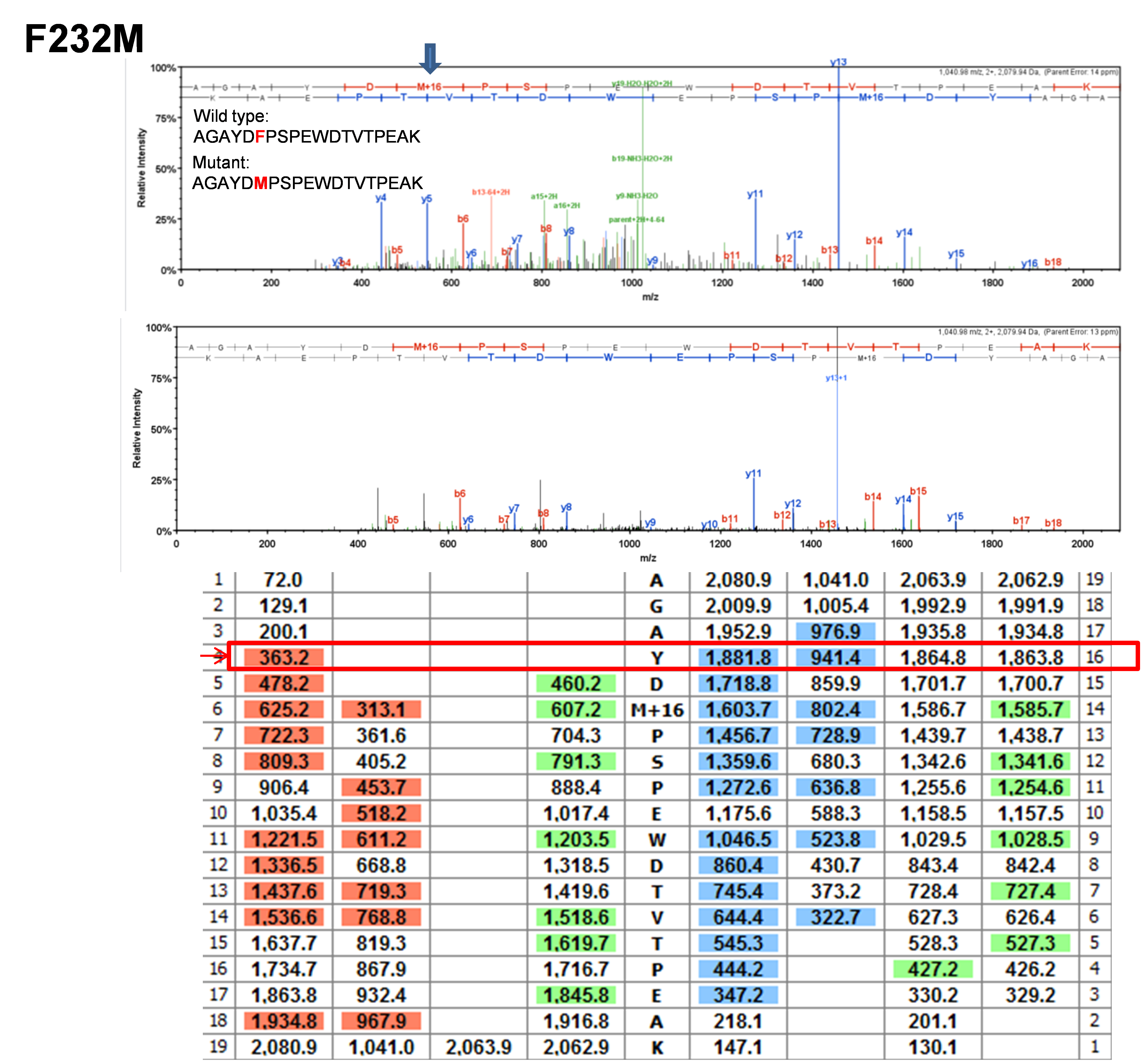

Supplement: S5 Fig — Spectra are shown from two independent experiments. (TIF) [file pgen.1005745.s005.tif]

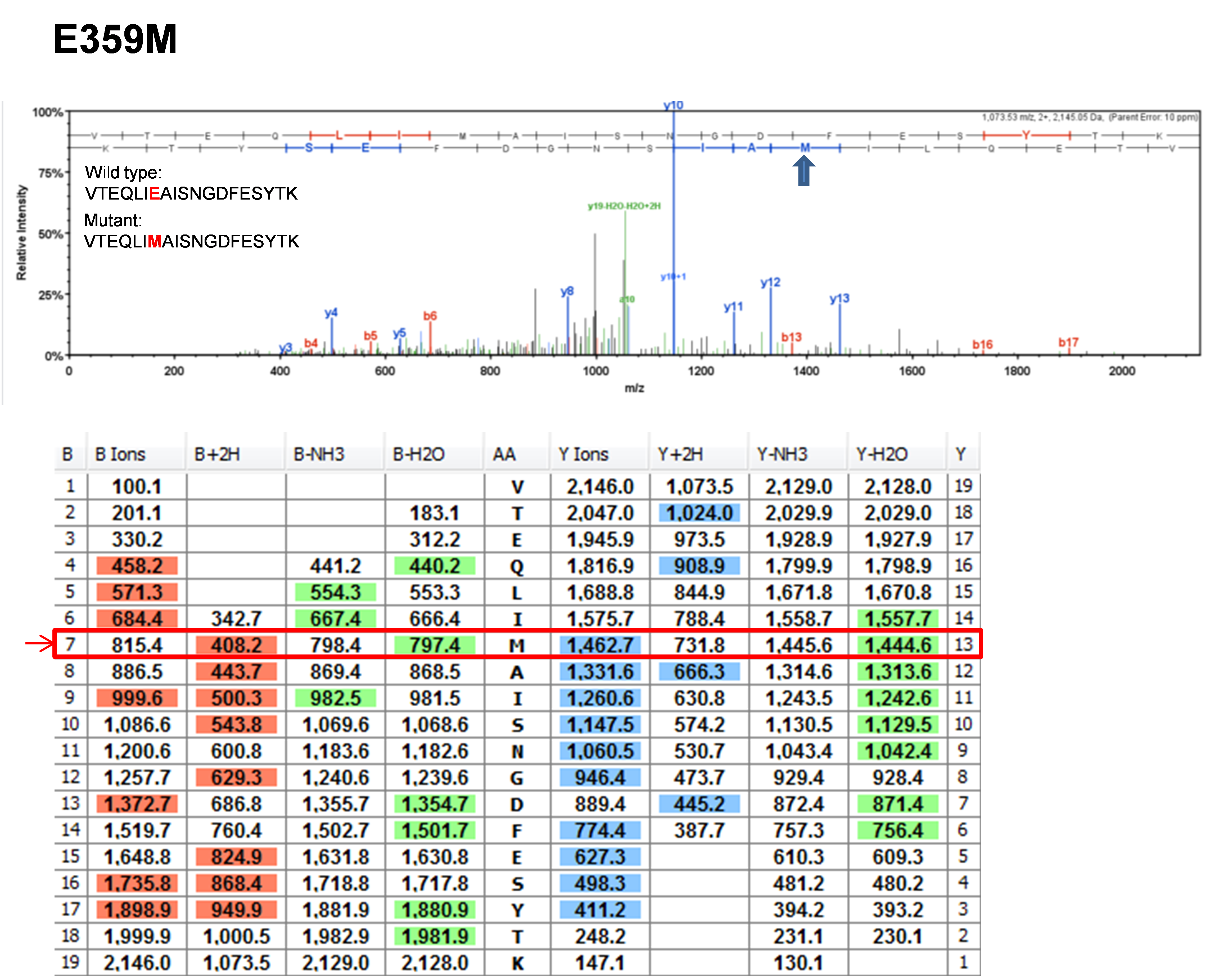

Supplement: S6 Fig — (TIF) [file pgen.1005745.s006.tif]

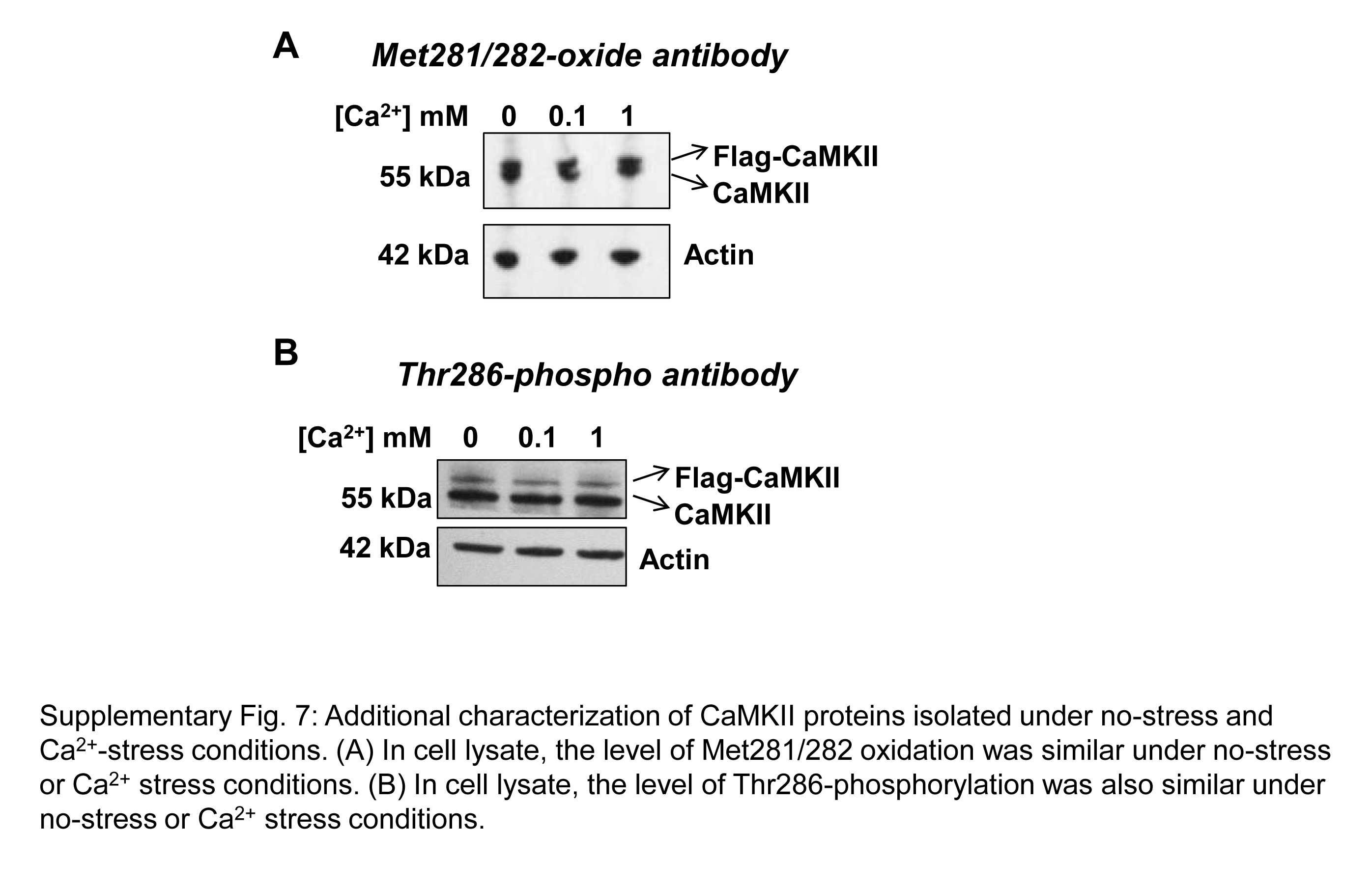

Supplement: S7 Fig — (A) In cell lysate, the level of Met281/282 oxidation was similar under no-stress or Ca2+ stress conditions. (B) In cell lysate, the level of Thr286-phosphorylation was also similar under no-stress or Ca2+ stress conditions. (TIF) [file pgen.1005745.s007.tif]

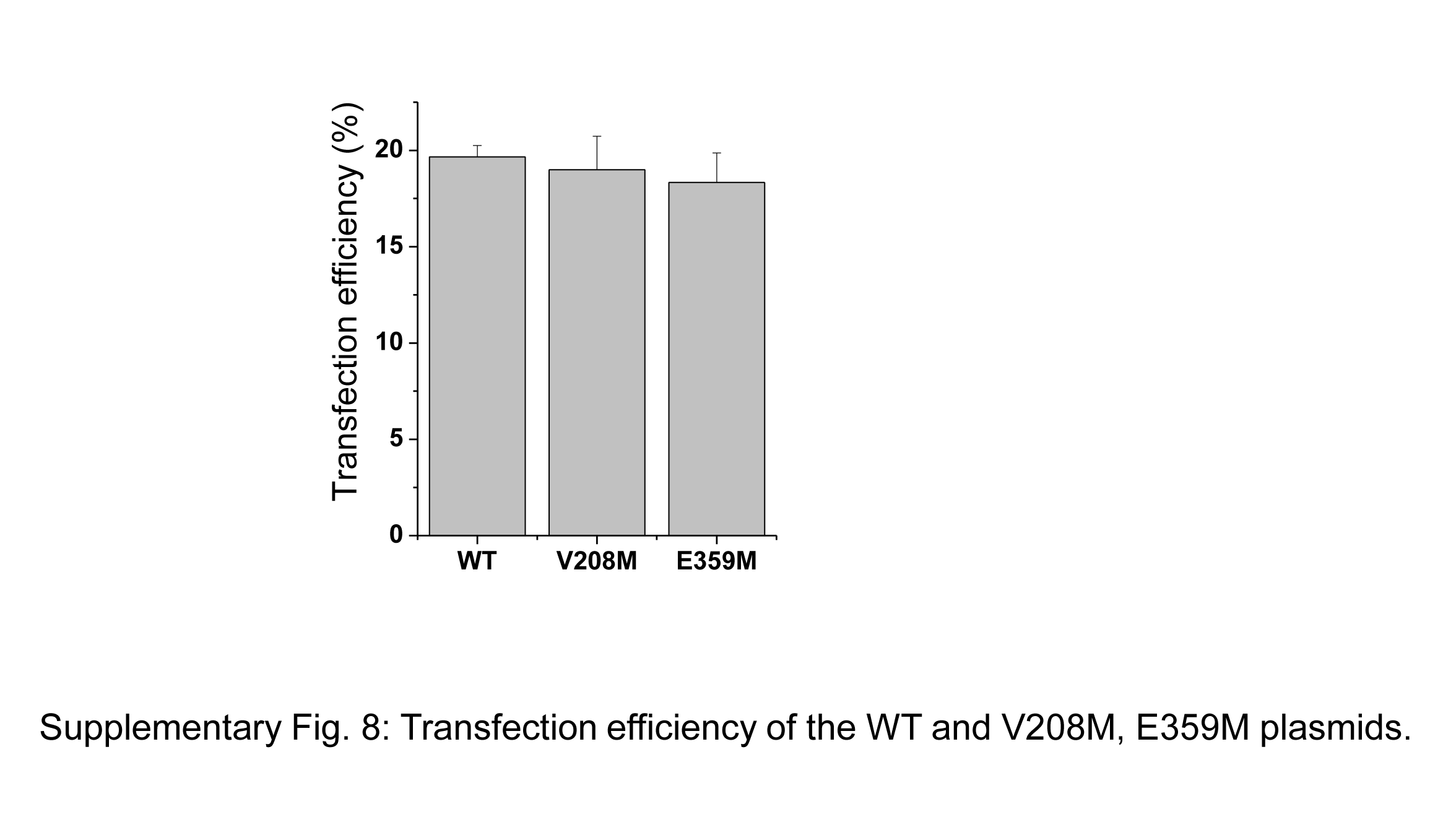

Supplement: S8 Fig — (TIF) [file pgen.1005745.s008.tif]

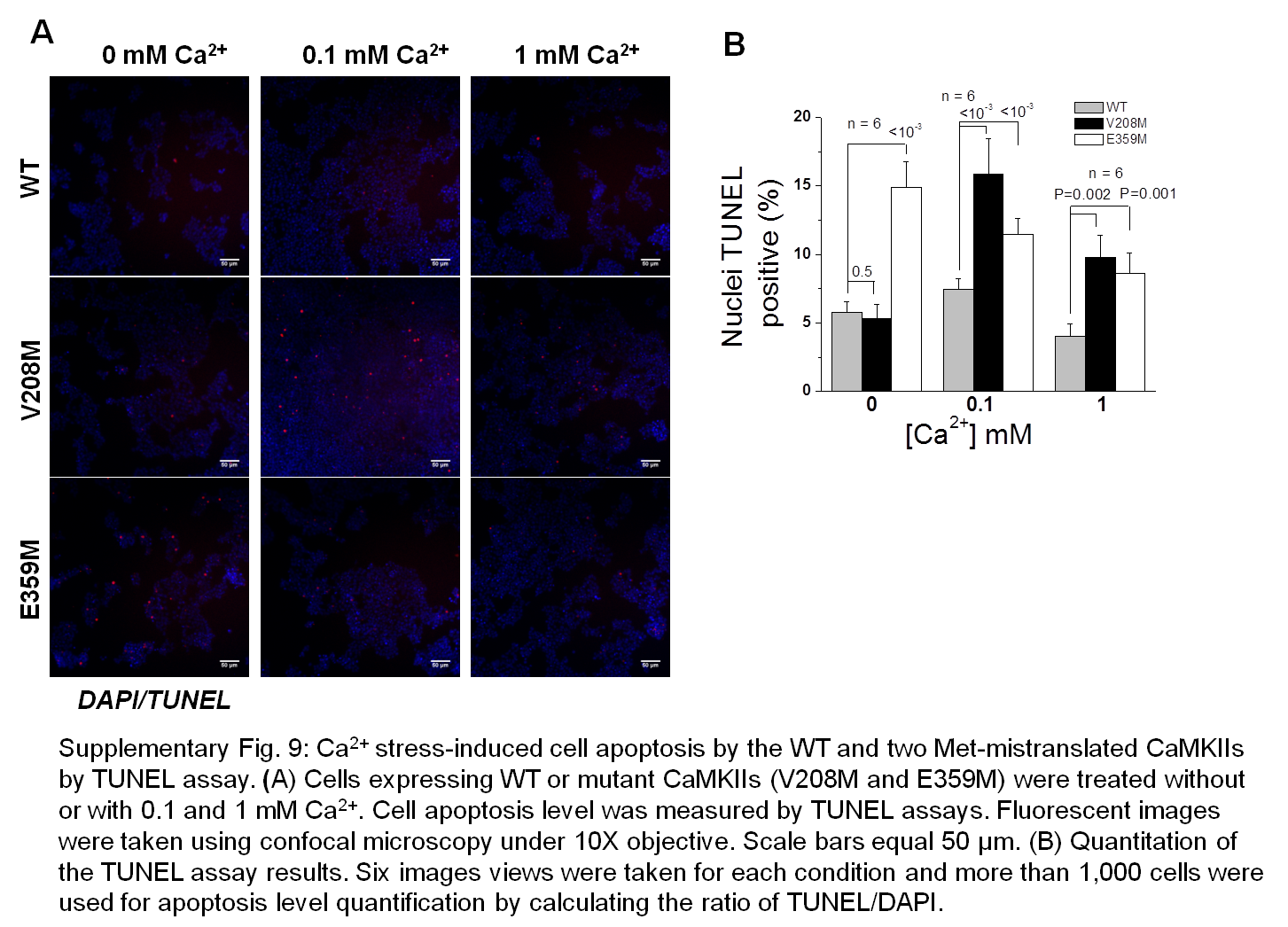

Supplement: S9 Fig — (A) Cells expressing WT or mutant CaMKIIs (V208M and E359M) were treated without or with 0.1 and 1 mM Ca2+. Cell apoptosis level was measured by TUNEL assays. Fluorescent images were taken using confocal microscopy under 10X objective. Scale bars equal 50 μm. (B) Quantitation of the TUNEL assay results. Six images views were taken for each condition and more than 1,000 cells were used for apoptosis level quantification by calculating the ratio of TUNEL/DAPI. (TIF) [file pgen.1005745.s009.tif]

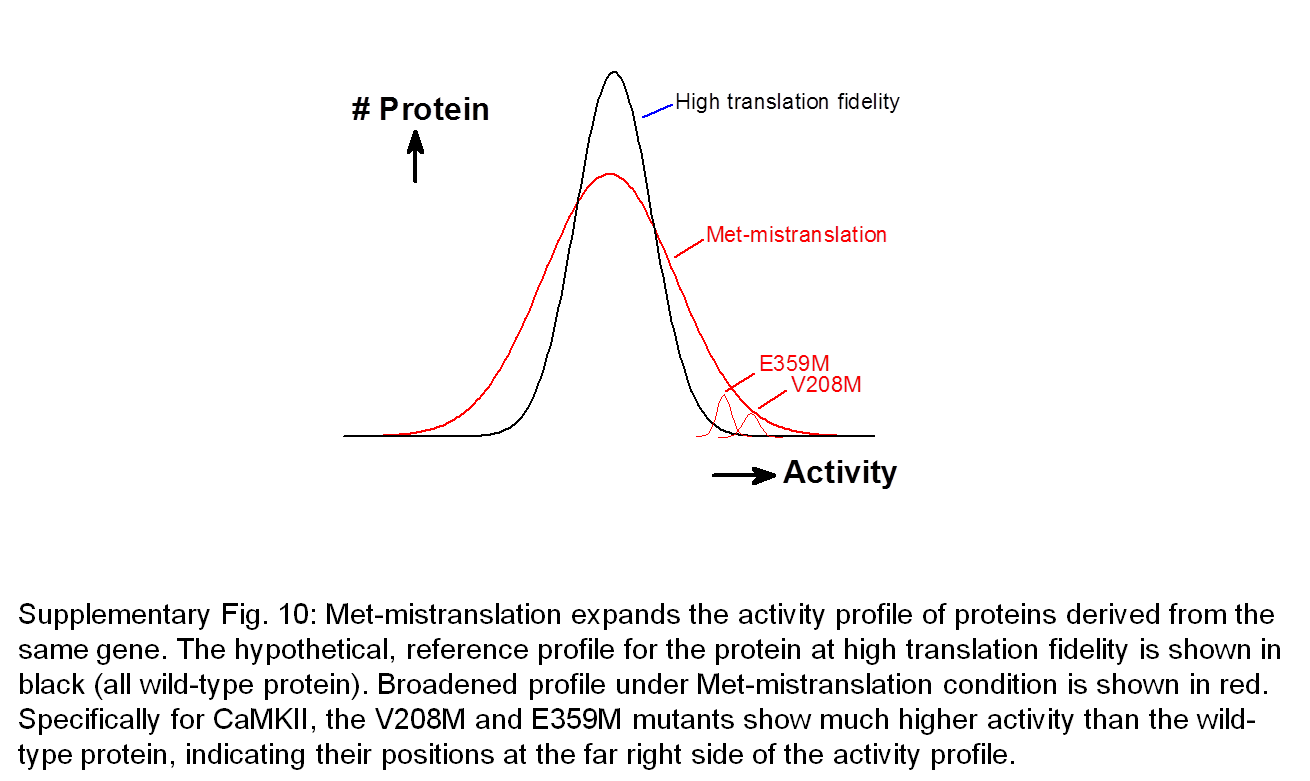

Supplement: S10 Fig — (TIF) [file pgen.1005745.s010.tif]
